# Evolutionary and structural aspects of Solanaceae RNases T2

**DOI:** 10.1590/1678-4685-GMB-2022-0115

**Published:** 2022-12-16

**Authors:** Claudia Elizabeth Thompson, Lauís Brisolara-Corrêa, Helen Nathalia Thompson, Hubert Stassen, Loreta Brandão de Freitas

**Affiliations:** 1 Universidade Federal de Ciências da Saúde de Porto Alegre, Departamento de Farmacociências, Porto Alegre, RS, Brazil.; 2 Universidade Federal do Rio Grande do Sul, Departamento de Genética, Porto Alegre, RS, Brazil.; 3 Universidade Federal do Rio Grande do Sul, Instituto de Química, Departamento de Fisico-Química, Laboratório de Química Teórica e Computacional, Porto Alegre, RS, Brazil.

**Keywords:** Ribonucleases T2, functional diversification, structural biology, Solanaceae, S-RNases

## Abstract

Plant RNases T2 are involved in several physiological and developmental processes, including inorganic phosphate starvation, senescence, wounding, defense against pathogens, and the self-incompatibility system. Solanaceae RNases form three main clades, one composed exclusively of S-RNases and two that include S-like RNases. We identified several positively selected amino acids located in highly flexible regions of these molecules, mainly close to the B1 and B2 substrate-binding sites in S-like RNases and the hypervariable regions of S-RNases. These differences between S- and S-like RNases in the flexibility of amino acids in substrate-binding regions are essential to understand the RNA-binding process. For example, in the S-like RNase NT, two positively selected amino acid residues (Tyr156 and Asn134) are located at the most flexible sites on the molecular surface. RNase NT is induced in response to tobacco mosaic virus infection; these sites may thus be regions of interaction with pathogen proteins or viral RNA. Differential selective pressures acting on plant ribonucleases have increased amino acid variability and, consequently, structural differences within and among S-like RNases and S-RNases that seem to be essential for these proteins play different functions.

## Introduction

The primary function of ribonucleases (RNases) is catalyzing the cleavage of RNA, hydrolyzing RNA to 3’-mononucleotides via 2’,3’-cyclic nucleotides. RNases are ubiquitous components of cells that act on single-stranded, double-stranded, or DNA-RNA hybrid substrates (Luhtala and Parker, 2010). Following the landmark crystallization of the bovine pancreatic RNase (Kunitz, 1940), many RNases have been described and characterized from a wealth of different species ranging from viruses to mammals. 

The transferase-type RNases are classified into three main families based on their molecular weight and specificity: RNase A, RNase T1, and RNase T2. These families differ in their pH optima, isoelectric points, and substrate specificities (Leeuw et al., 2007). RNase A primarily occurs in mammals; RNase T1 is commonly found in fungi and bacteria; and RNase T2 is widely distributed across kingdoms, with acidic pH optima (pH 4.0-5.0) and no specificity to nucleotide bases (Luhtala and Parker, 2010). More recently, Wu et al. (2020) showed that RNase T2 proteins preferentially cleave single-stranded RNA molecules between purine and uridine residues in human and other mammals, participating in several defense processes. 

The RNase T2 family plays a role in a range of biological functions, including the extracellular digestion of polyribonucleotides, remobilization of inorganic phosphate (Pi) from RNA under conditions of phosphate deficiency, protection against pathogens, prevention of self-fertilization, modulation of host immune responses, scavenging of nucleic acids, and degradation of self-RNA (Bariola et al., 1994; Leeuw et al., 2007; Luhtala and Parker, 2010). RNases T2 are classified based on their similarity to the *Aspergillus oryzae* RNase T2 (Kawata et al., 1998), which shows an adenylic acid preference without base specificity (Irie, 1999). The acidic activity of RNases T2 is compatible with their vacuolar or lysosomal localization. In most cases, RNase T2 proteins are secreted from the cell. Consequently, they are often glycosylated in eukaryotes (Deshpande and Shankar, 2002).

Plant RNases T2 are classified into three groups based on their function, sequence similarity, and genomic organization (Igić and Kohn, 2001); they are involved in various physiological and developmental processes (Green, 1994). Classes I and II comprise S-like RNases, acidic proteins with less than four introns in Class I and more than four in Class II (Igić and Kohn, 2001). In addition, experimental studies have shown that some S-like RNases exhibit a base preference (Kawano et al., 2002), whereas others have a broad base affinity (Kawano *et al.,* 2006). In addition, S-like RNases are induced in response to external stimuli, such as Pi starvation, senescence, wounding, and defense against pathogens (Kurata et al., 2002; Hayashi et al., 2003). 

On the other hand, Class III is composed of S-RNases, extracellular glycoproteins present in at least three plant families. Their RNase activity is related to the self-incompatibility (SI) system (Lee et al., 1994; Matton et al., 1994; Murfett et al., 1994; Hua et al., 2008; Pretz and Smith, 2022), a reproductive barrier that rejects genetically related pollens and accepts unrelated ones (Kubo et al., 2015; Ramanauskas and Igić, 2017). In most species displaying self-incompatibility, the discrimination between self- and non-self pollen is controlled by a multi-allelic *S*-locus (Torres-Rodríguez et al., 2020). Thus, variants of the *S*-locus are defined as haplotypes, and variants of the pollen or pistil components are called alleles (Chen et al., 2010). According to Iwano and Takayama (2012), two main types of SI can be observed in different plant families. That observed in Brassicaceae and Papaveraceae, which is based on a self-recognition system (Takayama and Isogai, 2005) with interactions between ligand and receptor molecules derived from a single S-haplotype; and that present in Plantaginaceae, Rosaceae, and Solanaceae that involves a non-self-recognition system (Iwano and Takayama, 2012; Aguiar et al., 2015) called S-RNase-based SI. In Solanaceae, the SI system is controlled by a pistil ribonuclease (S-RNase) with cytotoxic effects on self-pollen tubes, which degrades RNA and prevents pollination. Each S-locus haplotype encodes a single S-RNase (female S-determinant) and multiple S-locus F-box proteins (male S-determinants), whose interactions establish compatibility or incompatibility (Entani et al., 2014). While the RNA activity intrinsic to S-RNases is fundamental for their function, it is unknown how they inhibit the growth of self-pollen tubes. Experimental studies *in vitro* did not reveal any substrate specificity of S-RNases (Singh et al., 1991). However, they may behave differently *in vivo*.

Several reports on the phylogeny, biochemical properties, and three-dimensional X-ray diffraction structures of plant RNases T2 are available. However, few have included an extensive analysis of evolutionary aspects (MacIntosh et al., 2010) and their correlation with structural data. Despite recent advances in our understanding on the molecular mechanisms of S-RNase action, a more profound investigation into the molecular evolution of these proteins remains necessary. The molecular evolution of S-RNases, Class III RNases T2 in *Solanum* in the light of their three-dimensional structures has been described (Brisolara-Corrêa et al., 2015). 

The present investigation aims to understand the evolution and diversification of the Solanaceae RNase T2 family and the correlation with the structural variation found in the different classes. More specifically, our objectives are describing the evolutionary history of the RNase T2 genes in Solanaceae family and testing for positively selected amino acid residues as a signal of functional diversification. We carried out molecular dynamic simulations to analyze structural dissimilarities among and within the three classes of plant RNases T2. We hypothesized that evaluating the structural impact of specific amino acid replacements and studying 5’-GMP and 5’-AMP substrates complexed with S-RNases and S-like RNases would be of fundamental importance to understand functional and evolutionary aspects of Solanaceae RNase T2.

## Material and Methods

### Sequence data set

RNase T2 sequences were obtained from a Blastp (Altschul et al., 1990) search, which was conducted using the non-redundant protein database of the National Center for Biotechnology Information (NCBI) and Phytozome (Goldstein et al., 2012), with an S-like RNase sequence (RNase NE of *Nicotiana alata*, gi:532754) as a query. We downloaded 458 protein sequences of ribonucleases T2 and their nucleotide sequences from these repositories. We filtered the results for signals of pseudogenization (at least one premature stop codon in the coding region), significantly shorter length than average length (less than 300 nucleotides), and excluded sequences that did not include the two most highly conserved domains of RNases T2. After that, we retained 149 RNase sequences (Table S1). 

### Phylogenetic analyses of Solanaceae RNases T2

We performed multiple alignments for protein sequences in the COBALT webserver (Papadopoulos and Agarwala, 2007) using default parameters. Alignments were visually inspected and manually adjusted using MEGA X (Kumar et al., 2018). Phylogenetic trees were also visualized and edited with MEGA. All alignments are freely available upon request from the corresponding author.

We used jModelTest 2 (Darriba et al., 2012) and ProtTest 3 (Darriba *et al.,* 2011) to evaluate the best-fit evolutionary models for DNA and protein sequences, respectively. We based on the Akaike information criterion (AIC), Bayesian information criterion (BIC), and maximum likelihood (ML) tests to select the best substitution models. The distance method was applied using the Neighbor-Joining (NJ) algorithm, the p-distance matrix, pairwise deletion to treat gaps, and 1000 bootstrap replicates (Felsenstein, 1985). Maximum likelihood analysis was performed on the PhyML 3.0 webserver (Guindon et al., 2010). This tool calculates an initial BIONJ tree, with the parameters related to the proportion of invariable sites, the gamma distribution, and the empirical frequencies of amino acids and nucleotides estimated from the data. Four substitution rate categories were used, and branch lengths and parameters of the substitution model were optimized. Finally, the Subtree Pruning and Regrafting (SPR) algorithm was adopted to optimize the tree topology and an approximate likelihood ratio test (aLRT; Anisimova and Gascuel, 2006) was applied to calculate branches’ support. 

### Maximum likelihood tests of positive selection

Genes are subjected to different selective pressures, and many statistical methods have been developed to estimate them (Xu and Yang, 2013). The RNase T2 genes were analyzed based on models in which substitution rates (ω = d_N_/d_S_) varies among sites. Considering that synonymous changes are almost neutral, selection can be estimated comparing the rates of non-synonymous (d_N_) and synonymous changes (d_S_). A d_N_/d_S_ < 1 indicates purifying selection acting on the sites, whereas d_N_/d_S_ > 1 shows the positive selection action. The substitution rates and statistical parameters were calculated using the *codeml* tool in the PAML package (Xu and Yang, 2013). This tool permits comparing pairs of nested models with and without positive selection under a likelihood ratio framework. We applied two pairs of site-evolution models to test whether some sites (codons) are under positive selection: (1) the M1a (Nearly Neutral) that allows two site classes (0 < ω_0_ < 1 or ω_1_ = 1) and M2a (Positive Selection) that has an additional site class (ω > 1); and (2) the M7 (β), which allows 10 site classes with ω < 1, and M8 (β and ω) with one additional class allowing ω > 1 (Anisimova et al., 2001; Wong et al., 2004; Bielawski and Yang, 2005). Multiple alignments, including several different species, increase the power of detection of these methods due to the possibility to identify repeated substitution patterns in independent lineages (Ellegren, 2008).

Considering that most selection pressure is episodic, occurring at some particular moment along the evolutionary history, d_N_/d_S_ ratio should be statistically higher in lineages that have undergone positive selection than in other lineages. Consequently, the branch-site test can be used to determine if any of them show site-specific adaptations comparing dataset under null and alternative models. The tree branches were divided *a priori* into the foreground and background categories. There was a class of conserved sites with 0 < ω < 1 and a neutral class with ω_1_ = 1 along the background lineages. Also, a proportion (1 - p_0_ - p_1_) of sites corresponded to a selection with ω_2_ ≥ 1 in the foreground lineages. The ML tree topology and foreground branch were used, whereas branch lengths and other parameters were estimated by maximum likelihood under each model. A likelihood ratio test (LRT) was applied to verify if the null hypothesis is statistically different from the alternative one, which allows some sites under positive selection. The LRT compares twice the log-likelihood difference between the alternative and null model (2Δ*l*) to critical values from a χ^2^ distribution with degrees of freedom equal to the difference in the number of estimated parameters between both models. 

All indel (insertion-deletion) events represented by gaps in the alignments were considered in the pairwise comparisons. Finally, the Naive Empirical Bayes (NEB) and the Bayes Empirical Bayes (BEB) approaches were used to calculate the posterior probability (PP) of each site belonging to the site class of positive selection within each alternative model. 

### Molecular dynamics simulation

Four structures obtained by X-ray crystallography (PDB IDs: 1IOO, 1DIX, 1IYB, 1VD1) were submitted to molecular dynamics simulation using the GROMACS 4.5.5 package (van der Spoel et al., 2005) and the GROMOS96 53a6 force field (Oostenbrink et al., 2004). They represent clades 2 and 3 and subclades 2 and 3 of clade 2 of the phylogenetic tree. Structures were solvated with the Simple Point Charge model (SPC; Berendsen et al., 1987) for water molecules. Finally, counter ions (Na^+^ or Cl^-^) were added to neutralize the total protein charges.

The systems were submitted to energy minimization using the Limited-memory Broyde-Fletcher-Goldfarb-Shanno (LBFGS) algorithm and, subsequently, equilibrated in canonical (NVT) and isothermal-isobaric (NPT) ensembles for 0.5 nanoseconds (ns) each. Afterward, we performed molecular dynamics (MD) simulations of 119 ns in the NPT ensemble. All simulations were carried out at a constant pressure of 1 bar using the Parrinello-Rahman pressure-coupling scheme (Parrinello and Rahman, 1981) and a temperature of 298.15 K maintained by the V-rescale algorithm (Bussi et al., 2007). All protein bonds and water molecules were constrained using the Linear Constraint Solver (LINCS; Hess et al., 1997) and SETTLE algorithms (Miyamoto and Kollman, 1992). Coulomb and van der Waals interactions were computed within spherical cutoff radii of 1.25 nm. The smooth particle mesh Ewald method (SPME; Essmann et al., 1995) was applied to treat the long-range electrostatic interactions. Finally, the equations of motions were integrated using a time-step of two femtosecond (fs). Finally, the energy terms and densities of the systems were monitored and considered stable along the simulated trajectories. 

Employing utilities of the GROMACS package, we performed analyses of the mean root square deviation (RMSD, C-alpha), relative flexibility (root mean square fluctuation - RMSF; C-alpha, over the last five ns of all trajectories), secondary structure (using the DSSP tool; Touw et al., 2015), and solvent accessible surface area (SAS). The DSSP analysis resulted in a graphic showing the amino acid residues number against the time simulation that allowed to observe the protein secondary structure elements at each moment. The RMSD for pairwise comparisons of all modeled structures was calculated with the PyMOL 1.3software, which was also used to display and visualize the three-dimensional structures. The PyMOL plug-ins APBS (Adaptive Poisson-Boltzmann Solver; Baker et al., 2001) and PDB2PQR (Dolinsky et al., 2004) were used to calculate the electrostatic potential of S-like RNases and S-RNases.

The PDB files of 5’-GMP and 5’-AMP substrates available from 1IYB (RNase NW; Kawano et al., 2002) and 1VD1 (RNase NT; Kawano *et al.,* 2006) were complexed with 1DIX (RNase LE; Tanaka et al., 2000) and 1IOO (S-RNase; Ida et al., 2001) by superposition of their three-dimensional structures using PyMOL. Additionally, substrate 5’-GMP of 1IYB was complexed with the structure of 1VD1, and the substrate 5’-AMP of 1VD1 was complexed with the 1IYB three-dimensional structure. 

## Results

### Data retrieval and phylogenetic analyses

The 149 RNase T2 analyzed sequences were obtained from 11 Solanaceae genera (Table S1): *Brugmansia* (two sequences), *Dunalia* (one), *Eriolarynx* (two), *Iochroma* (four), *Lycium* (34), *Nicotiana* (15), *Petunia* (12), *Physalis* (20), *Solanum* (52), *Vassobia* (two), and *Witheringia* (five). For the proteins, the best substitution model based on the AIC, BIC, and ML tests was the JTT+G+F. In contrast, the DNA sequences were better described by the TPM3uf+G model in all tests (Table S2). As TPM3uf+G was not available in PhyML software, we used the GTR+G model that was the second best. The LRT analysis indicated that the differences between the two models are not statistically significant (*P* > 0.05; Table S3). 

**Figure 1 -:**
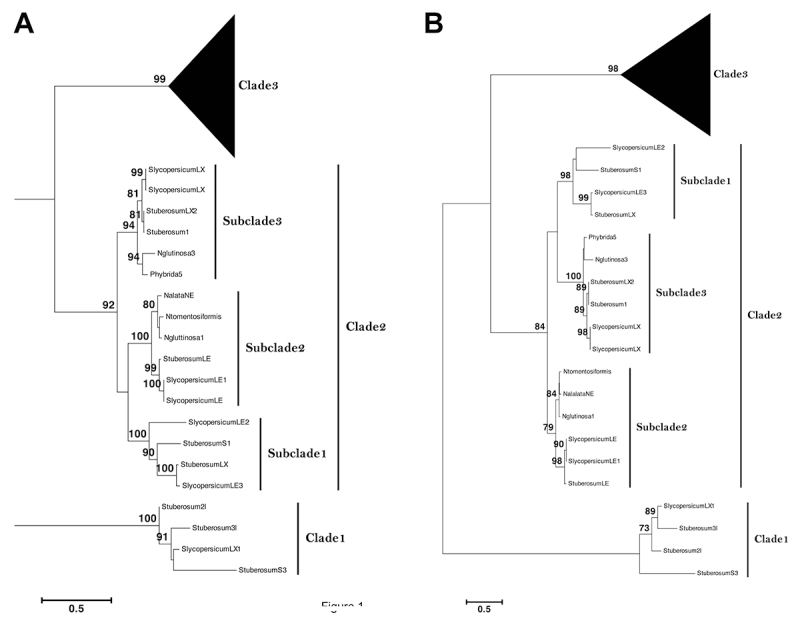
Maximum likelihood genealogies of Solanaceae RNases T2: (A) based on the coding nucleotide sequences; (B) based on deduced amino acid sequences. Numbers above the branches represent the branch support values. The scale bar indicates the levels of sequence divergence. Three clades and their subclades are labeled on the trees.

Both ML and NJ phylogenetic trees resulted in three statistically supported main clades for Solanaceae RNases T2 (Figures 1; S1 and S2). Clade 1 was composed of a widely divergent group of genes, all of them from the genus *Solanum*. According to GenBank, two (Slycopersicum_LX1 and Stuberosum_S3) were supported by expressed sequence tag (EST) evidence. Slycopersicum_LX1 is annotated as an intracellular ribonuclease LX-like; likewise, all other *Solanum* sequences in this clade are classified as S-like RNases. In contrast to clades 2 and 3, where proteins were characterized by acidic isoelectric points (pI), the proteins in clade 1 had a basic pI, except for Stuberosum_S3, which had a pI = 4.76 (Table S4).

For clade 2, we found three highly supported inner clusters, subclades 1, 2, and 3. Subclade 1 contained only sequences from *Solanum*; subclade 2 comprised two well-supported groups, one represented by sequences from *Solanum*, the other by sequences from *Nicotiana*. The third subclade was formed by one cluster of *Solanum* sequences and another containing *Nicotiana* and *Petunia* sequences. The *Solanum* sequences in clade 2 are annotated in GenBank as similar to S-like RNase LE or S-like RNase LX, S-like ribonuclease paralogs from the tomato (*S. lycopersicum*). 

Finally, clade 3 was formed by 10 subclades containing only S-RNase alleles (Figures 1 and S3). As there are more S-RNase than S-like RNase sequences in our databases for Solanaceae, clade 3 represented the largest sequence cluster. The evolution of the SI alleles was not compatible with the evolution of Solanaceae species. Subclade 1 contained three highly supported inner groups, 1A, 1B, and 1C. Groups 1A and 1B contained S-RNase sequences exclusively from *Lycium* and *Solanum*, respectively, while 1C contained sequences from the genera *Solanum*, *Lycium*, *Physalis*, *Nicotiana*, and *Petunia*. Subclade 2 was as big as subclade 1; it was formed by four highly supported monophyletic clusters, 2A (containing *Petunia* S-RNases), 2B and 2C (both containing sequences from the genus *Solanum*), and 2D (containing the genera *Physalis* and *Witheringia* sequences). The third subclade had two clusters: one (3A) composed of *Petunia* and *Lycium* S-RNases, and the other (3B) formed by *Solanum* sequences. The fourth subclade contained three groups: 4A (including *Brugmansia* and *Lycium* sequences), 4B (with *Witheringia*, *Physalis,* and a single sequence from *Brugmansia*), and 4C (containing *Vassobia*, *Nicotiana*, *Iochroma*, *Eriolarynx*, and *Dunalia* S-RNases). The fifth subclade was highly supported and exclusively held sequences from the genus *Solanum*. Subclade 6 consisted of *Solanum*, *Eriolarynx*, *Vassobia*, *Lycium*, and *Iochroma* sequences. Subclade 7 included *Solanum* and *Lycium* S-RNases, whereas subclade 8 contained sequences from *Petunia* and *Nicotiana*. The smaller subclade 9 was formed only by *Nicotiana* S-RNases. Finally, subclade 10 encompassed *Lycium* sequences. 

### Maximum likelihood tests of positive selection

**Figure 2 -:**
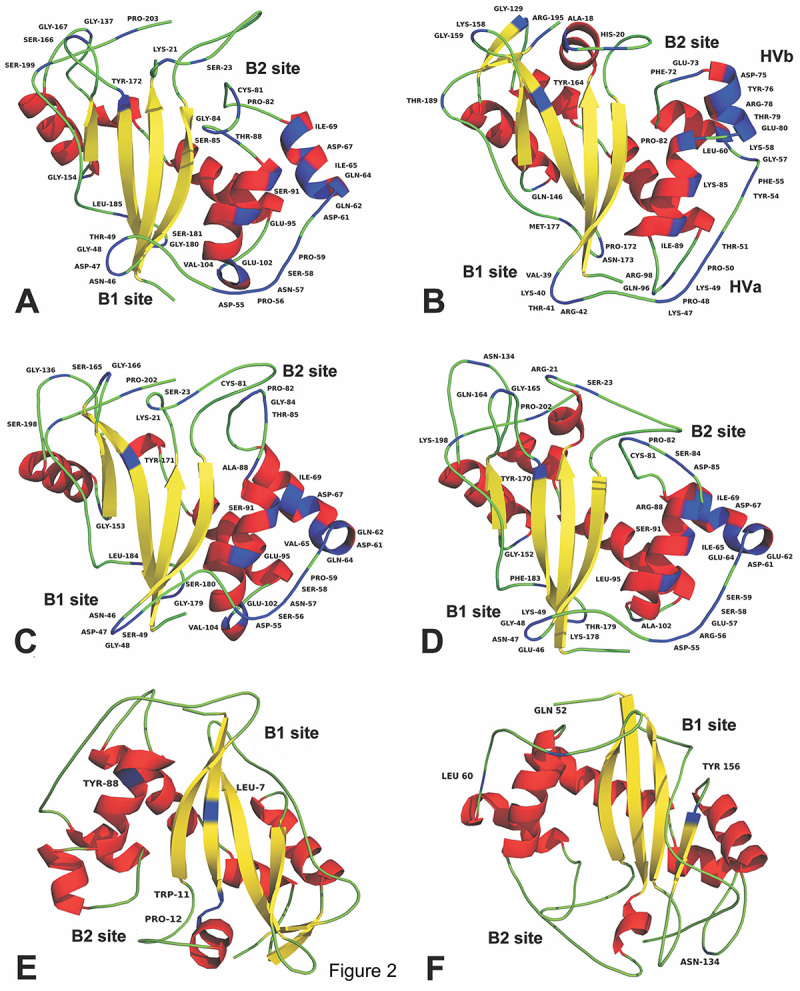
Three-dimensional structures from MD simulations highlighting the localization of the amino acid residues submitted to positive selection as identified by NSsites (A-D) and branch-site (E-F) tools of the PAML package for representative proteins: (A) 1DIX for subclade 2 of Clade 2; (B) 1IOO for Clade 3; (C) 1IYB for subclade 2 of Clade 2; (D) 1VD1 for subclade 3 of Clade 2; (E) 1IOO for Clade 3; and (F) 1VD1 for subclade 3 of Clade 2.

Our results indicated that, across Solanaceae T2 RNases, 41 amino acid residues were subject to positive selection (Table 1). A comparison of M1a (nearly neutral) and M2a (positive selection) furnished a significant LRT (494.1), thus rejecting the null hypothesis of neutrality (*p* < 0.0001). As a result, 1.5% of the sites with an ω = 2.2 and 31 amino acid residues were identified as being positively selected by BEB. The posterior probability was PP ≥ 99% for 27 of these. In our comparison of M7 (neutral, β) and M8 (selection, β and ω), the LRT was also significant (447.9; *p* < 0.0001). In addition, according to BEB, 1.7% of the sites with ω = 1.8 and 26 amino acids were subject to positive selection (23 with a PP ≥ 99%). Both model comparisons were highly significant, suggesting that positive selection was a relevant evolutionary force in the diversification of Solanaceae RNases T2. Most of the 31 positively selected amino acid residues detected by model M2a and BEB were predominantly located in coil/turn and helix regions of the proteins (Figures 2A-D).

**Table 1 -:** Parameter estimates, likelihood scores under models of variable ω ratios among sites, and positively selected sites (PSS) for T2 RNase genes in Solanaceae family.

Nested model pairs	lnL	2Δℓ (df, P-value)	dN/dS b	Parameter estimates c	PSSd BEB/NEB	BEB residues	NEB residues
Site-specific models							
M1a: nearly neutral (1)	-30055.37		0.13	*p*_*0*_ =0.9428, (*p*_*1*_ =0.0572) ω_0_=0.0796, (ω1=1.0000)	Not Allowed	---	---
M2a: positive selection (3)	-29808.34	494.06 (2, <0.0001)	0.20	*p*_*0*_ =0.9184, *p*_*1*_ =0.0663, (*p*_*2*_ =0.0153) ω_0_=0.1077, ω_1_=1.0000, ω_2_=2.2078	31 (27)/31 (26)	24, 26, 50, 51, 52, 58, 59, 61, 62, 63, 65, 66, 68, 70, 73, 76, 88, 89, 91, 92, 95, 96, 98, 101, 112, 114, 188, 195, 205, 206, 226	24, 26, 50, 51, 52, 58, 59, 61, 62, 63, 65, 66, 68, 70, 73, 76, 88, 89, 91, 92, 95, 96, 98, 101, 112, 114, 188, 195, 205, 206, 226
M7: β (2)	-29891.58		0.12	*p*=0.0291, *q*=0.2012	Not Allowed	---	---
M8: β & ω > 1 (4)	-29667.59	447.98 (2, <0.0001)	0.14	*p*_*0*_ =0.9823, (*p*_*1*_ =0.0177) *p*=0.0942, *q*=0.7463, ω=1.7906	26 (23)/41 (33)	24, 26, 51, 52, 58, 59, 61, 62, 63, 65, 66, 68, 73, 89, 91, 92, 95, 96, 101, 112, 114, 188, 195, 205, 206, 226	24, 26, 49, 50, 51, 52, 58, 59, 61, 62, 63, 65, 66, 68, 70, 71, 73, 74, 76, 88, 89, 91, 92, 94, 95, 96, 98, 101, 105, 112, 114, 150, 169, 188, 189, 195, 205, 206, 210, 226, 232

a The number after the model code, in parentheses, is the number of free parameters in the *ω* distribution.

b This d_N_/d_S_ ratio is an average over all sites in the T2 RNase gene alignment.

c Parameters in parentheses are not free parameters and are presented for clarity.

d PSS is the number of positive selection sites according Bayes Empirical Bayes (BEB) and Naive Empirical Bayes (NEB). The first number is the PSS with posterior probabilities ≥ 95%. The second number (in parentheses) is the PSS with posterior probabilities ≥ 99%.

Having established that Solanaceae RNases T2 evolved under positive selection, we evaluated whether molecular evolution was accelerated in particular lineages. The branch-site test indicated that the lineages represented by subclade 3 (foreground branch 3) of clade 2 and by clade 3 (foreground branch 6) were subjected to diversifying selection (Table S5). Subclade 3 was formed by two groups, one containing only *Solanum* and another with *Nicotiana* and *Petunia* S-like RNases (Figure 1). This subclade had three amino acids under positive selection according to BEB. In clade 3, one amino acid residue was positively selected according to BEB; considering NEB, this increased to four. Positively selected residues were predominantly located in the coil/turn regions of the proteins from clade 3 and clade 2-subclade 3 (Figures 2E and F).

### Structural analyses

S-RNase and S-like RNase proteins submitted to molecular dynamics simulations belong to the (α + β) class and possess an antiparallel β-sheet in their center (Figure 2). Details of the number of helices and strands, catalytic residues, base binding sites, and disulfide bonds identified by the GROMACS program are available in Table 2. 

**Table 2 -:** Structural information about the S-RNase and S-like RNases submitted to molecular dynamics simulation.

PDB ID	RNase type	Function	α-helices	β-strands	Catalytic residues	Base binding sites	Disulfide bonds
1DIX	S-like RNase	Response to tissue damage or protection against pathogens	α1: 15 to 17 αA: 62-67 αB: 69-75 αC: 85-96 αD: 108-121 αE: 124-130 αF: 142-153	β1: 4-12 β2: 37-44 β3: 139-141 β4: 158-163 β5: 169-179 β6: 185-186 β7: 199-201	His39 His92 His97	B1: Tyr8, Trp42, Tyr50, Tyr175 B2: Tyr17, Phe89, Tyr172, Leu79, Thr78	Cys18-Cys24 Cys25-Cys81 Cys54-Cys100 Cys161-Cys196 Cys177-Cys188
1IOO	S-RNase	Self-incompatibity system	α1: 11-19 α2: 56-67 α3: 73-81 α4: 81-91 α5: 92-95 α6: 100-114 α7: 116-124 α8: 133-146	β1: 4-10 β2: 30-37 β3: 130-132 β4: 150-154 β5: 162-170 β6: 177-178 β7: 192-194	His32 Tyr86 His91	B1: Tyr4, Gln6, Trp35, Asp37, Arg42 B2: Thr10, Phe15, Gln69, Leu70, Lys71, Phe72, Pro82, Ser83	Cys16-Cys21 Cys46-Cys94 Cys153-Cys186 Cys169-Cys180
1IYB	S-like RNase	Response to tissue damage or protection against pathogens	α1: 14-16 α2: 61-66 α3: 68-74 α4: 87-95 α5: 97-100 α6: 106-119 α7: 122-128 α8: 140-152	β1: 6-12 β2: 37-43 β3: 137-139 β4: 157-162 β5: 168-178 β6: 198-200	His39 His92 His97	B1: Trp42, Asn44, Tyr50 B2: Gln12, Tyr17, Thr78, Leu79, Phe89	Cys18-Cys24 Cys25-Cys81 Cys54-Cys100 Cys160-Cys195 Cys176-Cys187
1VD1	S-like RNase	Response to tobacco mosaic virus infection (TMV)	α1: 14-16 α2: 62-75 α3: 87-95 α4: 98-100 α5: 105-119 α6: 121-128 α7: 140-151	β1: 6-12 β2: 37-43 β3: 133-139 β4: 156-162 β5: 167-176 β6: 183-185 β7: 198-201	His39 His92 His97	B1: Trp42, Asn44, Trp50 B2: Gln12, Tyr17, Ser78, Leu79, Phe89	Cys18-Cys24 Cys25-Cys81 Cys54-Cys100 Cys159-Cys195 Cys175-Cys186

**Figure 3 -:**
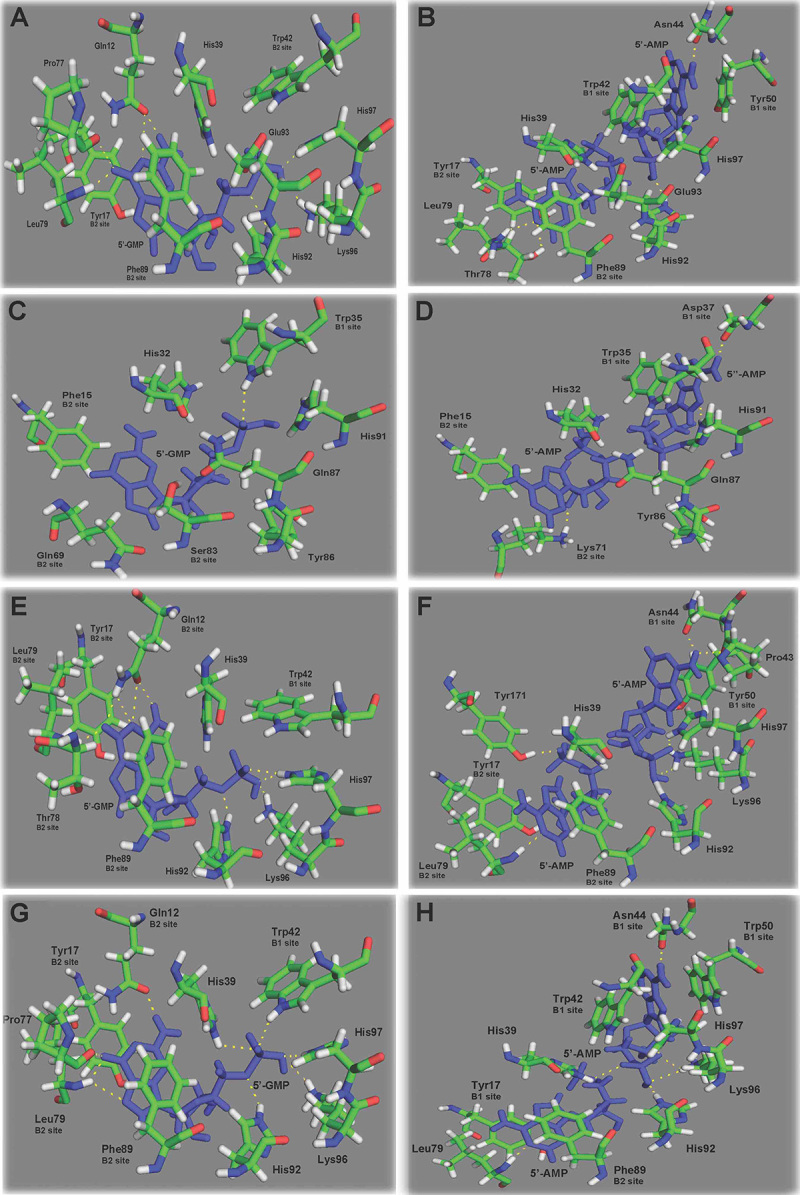
View of the substrate-binding site of RNase LE (PDB ID 1DIX) from cultivated tomato (*Solanum lycopersicum*) complexed with (A) 5’-GMP and (B) 5’-AMP, substrate-binding site of S-RNase (PDB ID 1IOO) from *Nicotiana alata* complexed with (C) 5’-GMP and (D) 5’-AMP, substrate-binding site of RNase NW (PDB ID 1IYB) from *Nicotiana glutinosa* complexed with (E) 5’-GMP and (F) 5’-AMP, and substrate-binding site of RNase NT (PDB ID 1VD1) from *Nicotiana glutinosa* complexed with (G) 5’-GMP and (H) 5’-AMP. Dashed lines indicate hydrogen bonds. The substrates are colored in blue.

The *Solanum lycopersicum* (LE) RNase (PDB ID 1DIX; clade 2, subclade 2) has a catalytic site composed of four residues (His92, Glu93, Lys96, and His97) in the αC-helix and two (His39 and Trp42) in the β2-strand (Figure 2A). In addition, it has two putative base-binding sites responsible for RNA degradation: B1, which is formed by Tyr8, Trp42, Tyr50, and Tyr175, and B2, a hydrophobic pocket formed by the side-chains of Tyr17, Phe89, and Tyr172 (Figures 3A and B). 

The S_F11_-S-RNase of *Nicotiana alata* (PDB ID 1IOO; clade 3) has a hydrophobic core constituted by Leu5, Leu7, Leu9, Ile31, Leu34, Ile151, Leu163, Ile166, and Phe170. Its catalytic site is formed by His32, Tyr86, and His91, while Trp35 is part of the B1 substrate-binding site, together with Tyr4, Gln6, Asp37, and Arg42 (Figure 2B). The B2 site comprises Thr10, Phe15, Gln69, Leu70, Lys71, Phe72, Pro82, and Ser83 (Figures 3C and D). S-RNases are involved in the SI system and have two crucial hypervariable regions (HVa and HVb) (Figure 2B). Several positively charged amino acids were found in S-RNase HVa: Lys40, Arg42, Lys47, Lys49, Lys85, and Lys90. The HVb region consists of the two-turn α-helix (α3) near HVb; the amino acids Glu73, Asp75, and Glu80 confer a negative charge to the protein. 

The *Nicotiana glutinosa* ribonuclease NW (1IYB; clade 2, subclade 2) features an extended helical region formed by α2-α3-α4-α5-α6-α7 (Figure 2C). Essential residues of its catalytic site are His39, His92, and His97. The B2 site is comprised of Gln12, Tyr17, Thr78, Leu79, and Phe89, whereas the highly conserved Trp42, Asn44, and Tyr50 amino acids form the B1 site (Figures 3E and F).

The three-dimensional structure of the *N. glutinosa* RNase NT (1VD1) obtained by Kawano et al. (2006) was included in the subclade 3 of clade 2. Like the S-like RNases described above, 1DIX and 1IYB, 1VD1 has three catalytic histidine residues in its catalytic site (His39, His92, and His97) (Figure 2D). Trp42, Asn44, and Trp50 form the B1 site, while the key residues contributing to the B2 site are Gln12, Tyr17, Ser78, Leu79, and Phe89 (Figures 3G and H).

**Figure 4 -:**
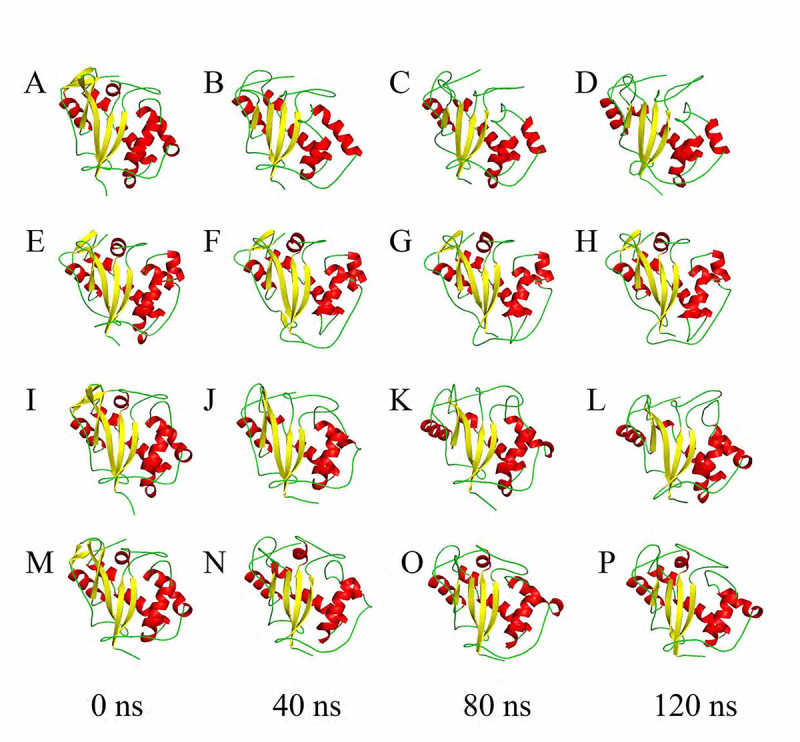
Three-dimensional structures of RNase LE (A-D), S-RNase (E-H), RNase NW (I-L), and RNase NT (M-P) after 0 ns, 40 ns, 80 ns, and 120 ns of molecular dynamics simulation.

These initial structures were submitted to molecular dynamics simulations for 120 ns. The RMSD curves (Figure S4) demonstrated that all structures converged to a constant value for the last 20 ns. Figure 4 illustrates the three-dimensional structures of 1DIX at 0 ns (A), 40 ns (B), 80 ns (C), and 120 ns (D) simulations and shows that there was a reduction in α-helix and turn content in the structure of 1DIX. The increase in the number of coils led to an overall decrease of defined structure elements, which were defined as the sum of α-helices, β-sheets, β-bridges, and turns (Figure S5A). These events produced an increase in the RMSD after 30 ns (Figure S4). For 1DIX, we observed that β1, β2, β3, β4, β5, β6, αD, αE, and αF were maintained throughout the simulation, whereas a β-bridge substituted β6, α1 was disrupted, and a single helix replaced αA and αB. αC became unstable during the simulation (Figure S6A). 

The secondary structure of the 1IOO S-RNase was maintained, except the β6-strand, which was converted into a β-bridge, similar to what happened in 1DIX (Figures 4E-H; S5B; and S6B). 

The RMSD for 1IYB increased sharply after 80 ns (Figure S4), which was correlated with a decrease in the number of residues involved in α-helices and turns and an increase in the coil content (Figure 4I-L; and S5C). From Figure S6C, we observe that β3, α1, and α2 were disrupted, β6 was converted into a β-bridge, and a β-strand was formed between residues 191 and 193. Additionally, α4, α5, and α7 were unstable during the simulation. The number of residues in β-sheets increased after 70 ns, increasing secondary structure content (Figure S5C). 

For the 1VD1 RNase, molecular dynamics analyses showed the formation of β3 and β7 around 80 ns and their disruption near 90 ns (Figures S5D and S6D). These β-bridges form a two-stranded antiparallel β-sheet away from the central core of the protein. In contrast to 1DIX and 1IYB, the structures of 1IOO and 1VD1 present minor relative mobility during the last five ns of MD simulation as evidenced by the RMSFs (Figure S7). The S-like RNase structures 1DIX, 1IYB, and 1VD1 (Figure S8A, C, and D, respectively) possessed a larger hydrophilic solvent accessible surface (SAS). In contrast, 1IOO, which represented S-RNases, was more hydrophobic (Figure S8B).

All amino acids identified as positively selected by NSsites were located in the four final structures derived from MD analyses (Figures 2A-D). Additionally, the branch-site analysis indicated that clade 3 and subclade 3 of clade 2 experienced positive selection. This positive selection may indicate that diversification, including the evolution of different specificities, occurs through substitutions in different regions of S-RNases and S-like RNases in different lineages of the tree. The amino acids identified by branch-site analysis are highlighted in Figures 2E and F. 

In 1DIX (subclade 3 of clade 2), NSsites identified three regions of positively selected amino acids. The RMSF curves exhibited increased mobility (Figure S7A). They were located in coil (Pro56, Asn57, Ser58, and Pro59), bend (Ser166 and Gly167), and turn (Gly180 and Ser181) elements (Figure 2A). For the S-RNase 1IOO, the main region containing the positively selected amino acids Lys158 and Gly159 was responsible for considerable mobility (Figure 2B and S7B). Interestingly, the sites identified by the branch-site test (Leu7, Trp11, Pro12, and Tyr88; see Figure 2E) were in the least flexible regions of this S-RNase. In 1IYB, the highest mobility was located in a bend region that contained the positively selected amino acids Pro82, Gly84, and Thr85. In addition, two other bend regions represented by the Asp47, Gly48, Ser49, Ser165, and Gly166 residues were highly flexible (see Figures 2C and S7C). In the structure of 1VD1, there were at least four positively selected amino acids with increased mobility (Asn47, Glu57, Asp61, and Glu62) in a coil between β2 and α2. Gln164 was also in a flexible coil between β4 and β5. Gln52, which was found to be positively selected in the branch-site test, is located in a region with increased mobility (Figures 2D and F; and S7D). 

The distinct structural conformation of S-like RNases near the B2 substrate-binding site is worth noting. Compared to S-RNases, they showed increased flexibility, achieved by an elevated content of coils near residue numbers 21-23 and 80-85 (Figures 2A, C, and D). Additionally, several positively selected residues (Table 1) were close to Phe89, an essential hydrophobic amino acid for the B2 substrate-binding site in S-like RNases (Table 2; Figures 2 and 3). S-like RNases furthermore had a positively selected tyrosine residue near position 170, again close to the B2 site (Figures 2A, C, and D). The differences between S-like RNases and S-RNases were less pronounced at the B1 binding site (Figure 2). The tests for positive selection found that several residues in this region had a significantly elevated number of non-synonymous substitutions; these were residues 45-49 in S-like RNases (Figures 2A, C, and D), and residues 39-42 in S-RNases (Figure 2B). Other regions of structural importance containing several positively selected amino acids were found in the hypervariable regions of S-RNases (Figure 2B).

The amino acid residues identified as site-specific adaptations in the lineages formed by clade 3 and subclade 3 of clade 2 were located in distinct regions of the representative proteins (Figure 2E and F). In S-RNases, the residues Leu7 near the B1 substrate-binding site and Trp11 and Pro12 near B2 showed low mobility as demonstrated by the RMSF analysis (Figure S7B). This rigidity may play an essential role in RNA degradation. For example, in the S-like RNase 1VD1, the highly mobile Gln52 was near the B1 site, whereas Leu60 was close to the catalytic residue His92. The differences between S-RNases and S-like RNases in the flexibility of amino acids near the substrate-binding region are likely to hold vital clues to better understand the RNA binding process. On the other hand, two positively selected amino acid residues (Tyr156 and Asn134) found in S-like RNases were located in very flexible regions of the molecular surface. Considering that the S-like RNase 1VD1 is induced to respond to the tobacco mosaic virus infection, these may be the regions of interactions with the pathogen proteins.

**Figure 5 -:**
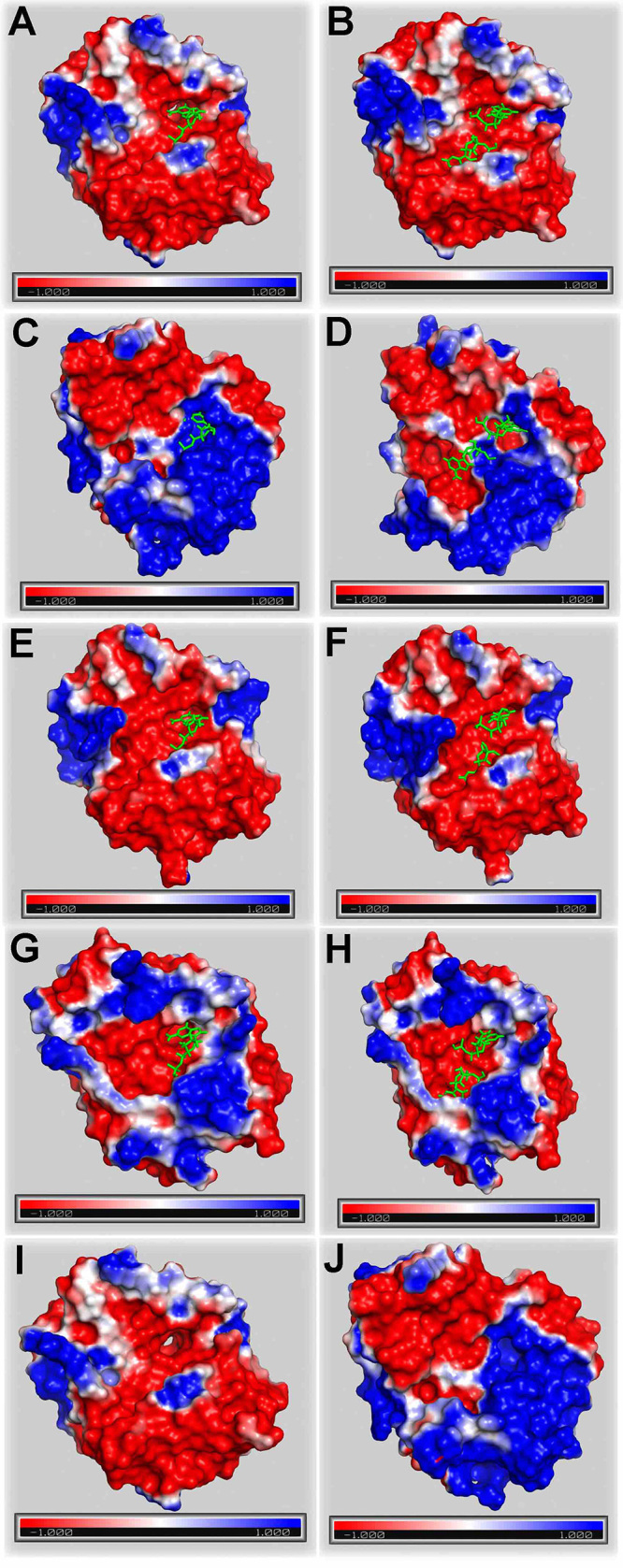
Mapping the electrostatic potential at the molecular surface of S-like RNases and S-RNases. 1DIX complexed with (A) 5’-GMP and (B) 5’-AMP. 1IOO complexed with (C) 5’-GMP and (D) 5’-AMP. 1IYB complexed with (E) 5’-GMP and (F) 5’-AMP. 1VD1 complexed with (G) 5’-GMP and (H) 5’-AMP. Three-dimensional structures of 1DIX (I) and 1IOO (J) without substrates. The substrates are colored in green.

The three-dimensional structure superposition of an S-RNase (1IOO) and three S-like RNases (1DIX, 1IYB, and 1VD1) allowed the identification of key catalytic residues of S-RNases (Figures S9A-C): His32, Tyr86, and His91. The catalytic and B1 and B2 base-binding sites are fundamental for RNA recognition and cleavage. We constructed complexes of the three S-like RNases and the S-RNase with their 5’-GMP and 5’-AMP substrates (Figure 3). In the complex of RNase LE (1DIX) and 5’-GMP (Figure 3A), we identified a critical stacking interaction between the amino acids Tyr17 and Phe89 and the substrate occupying the B2 binding site. Leu90, His92, Lys96, and His97 established hydrogen bonds with the substrate. On the other hand, while occupying the B1 site, 5’-AMP (Figure 3B) was stabilized by hydrogen bonds with Asn44 and stacking interactions with Trp42 and Tyr50. Tyr17 and Phe89 also seemed to play an essential role in binding 5’-AMP to the B2 site, as did Thr78, Leu90, and His92, which all formed hydrogen bonds with 5’-AMP. The three-dimensional structure of RNase NW (1IYB) complexed with 5’-GMP is available in Protein Data Bank. Stacking interactions between residues Tyr17 and Phe89 and the RNA base in the B2 site could be seen for the complex of RNase NW with 5’-GMP (Figure 3E) or 5’-AMP (Figure 3F) and the complex of RNase NT (1VD1) with 5’-GMP (Figure 3G) or 5’-AMP (Figure 3H). Near Tyr17, there was a positively selected amino acid (Tyr171). The complex of RNase NW with 5’-GMP was stabilized by hydrogen bonds involving Gln12, Thr78, His92, Lys96, and His97 (Figure 3E), with 5’-AMP by hydrogen bonds with Pro43, Asn44, Leu79, Lys96, His97, and Tyr171 (Figure 3F). The complex of RNase NT and 5’-AMP (Figure 3H) also showed stacking interactions at the B2 binding site. We found complexes of S-RNase with 5’-GMP stabilized by a single hydrogen bond involving Trp35 (Figure 3C), and there was no stacking sandwich-like found in S-like RNases (Tyr17-substrate-Phe89). This difference could be found due to a serine residue occupying the position of Phe89 in the B2 binding site near 5’-GMP in S-RNases. Serine is a polar and hydrophilic amino acid. Additionally, two positively selected residues (Phe72 and Pro82) were located near Gln69 in the B2 site. When complexed with 5’-AMP (Figure 3D), S-RNase also lost the stacking interactions at both B1 and B2 binding sites, forming a significantly smaller number of hydrogen bonds with the substrate.

The electrostatic potential mapped on the molecular surface of S-like RNases and S-RNases is depicted in Figure 5. The most remarkable similarity was found between RNase LE (1DIX; Figures 5A and B) and RNase NW (1IYB; Figures 5E and F). The 5’-GMP substrate seemed to fit similarly into the B2 binding site of the three-dimensional structures of RNases LE (Figure 5A), NW (Figure 5E), and NT (Figure 5G); since they all cleave guanylic acid, this was not surprising. The electrostatic potential was also similar for these S-like RNases in that region. Therefore, it is interesting to note the positive electrostatic potential at the S-RNase B2 site (Figure 5C) in the complex with 5’-GMP. It is known that RNase NT (1VD1) can also hydrolyze Poly A; its electrostatic potential is shown in Figure 5H. The electrostatic potential of the S-RNase complexed with 5’-AMP (Figure 5D) was more negative than when complexed with 5’-GMP (Figure 5C). The electrostatic potentials of RNase LE and S-RNase outside of substrate complexes are shown in Figures 5I and J, respectively. 

## Discussion

The biological function of a protein is highly dependent on its three-dimensional conformation. Although proteins usually exist in a single native state, they are subjected to different evolutionary forces that might favor specific amino acid substitutions and, thus, lead to essential variations in the active site or regions of monomer-monomer interactions, for example. These modifications commonly have an impact on protein function. For example, gene duplication is one of the primary mechanisms leading to the emergence of new genes. Accelerated non-synonymous substitution rates can provide unique features selected in a proper environment. Phylogeny-based models of codon substitution represent valuable tools for studying adaptation at the molecular level, thus complementing structural and biochemical studies (Bishop, 2005). 

This study analyzed the evolutionary history of S-RNases and S-like RNases from the Solanaceae family. The experimentally determined crystal structures of RNases T2 were analyzed, and residues found to be under positive selection were considered in the context of their location in relation to important structural features, as well as their physical and chemical properties. The S-RNase genes involved in the self-incompatibility system do not present a phylogenetic signal when considering the relationships among sequences within Solanaceae. Instead, they diverged according to the different botanical families, which are explained by the long divergence time between species from suprageneric taxonomic entities (Brisolara-Corrêa et al., 2015, for review). 

We identified three highly supported inner clusters in clade 2, subclades 1, 2, and 3. First, the involvement of RNase LX in the Pi starvation response in tomato plants has been demonstrated (Köch et al., 2006). Phosphate plays an essential role in primary metabolism. When phosphate supply is low, scavenging of Pi from macromolecules is induced to sustain phosphate-dependent processes. Moreover, the expression of RNase LX is increased after ethylene treatment, suggesting a connection with senescence processes (Lers et al., 1998). RNase LE seems to play a role in responding to mechanical wounding and tissue damage, such as the protection against pathogens (Kurata et al., 2002). These authors identified in *Nicotiana glutinosa* a wound-inducible ribonuclease (RNase NW, PDB ID 1IYB) and an S-like RNase with high similarity to RNase LX (RNase NT, PDB ID 1VD1). RNase NT is significantly induced after 48 h in response to an infection with the tobacco mosaic virus (TMV); it is thus possibly a defensive protein. The *Nicotiana alata* S-like RNase (RNase NW) is also induced under phosphate-limited conditions (Dodds et al., 1996). 

Clade 1, the most divergent group, includes *Solanum* sequences. Some of these are supported by expressed sequence tag (EST) evidence according to GenBank (Slycopersicum_LX1 and Stuberosum_S3) and annotated as intracellular ribonucleases. Of note, this cluster is formed by proteins with a basic pI, except for Stuberosum_S3, which has a pI of 4.76 (Table S4). 

Hillwig et al. (2010) have also described three main clades representing three RNase T2 classes, in addition to four *Petunia* S-like RNases that exhibit unique expression patterns in different plant tissues. MacIntosh et al. (2010) have identified three main clades in their analysis of plant RNases T2. Along with several sequences from rice, they included seven Solanaceae sequences and obtained a cluster labeled Class I with an inner group represented by *Solanum*. This subclade corresponds to our subclade 2 of Clade 2. These studies indicate that S-RNases and S-like RNases share a common ancestor. Previous studies have also found three classes of plant RNases T2 (Igić and Kohn, 2001: Steinbachs and Holsinger, 2002; Roalson and McCubbin, 2003). However, none of these studies included an extensive evolutionary analysis in the light of structural data. 

Differences in the non-synonymous rates are expected for proteins with some level of diversification. Examples are proteins involved in host-pathogen interactions, such as pathogenesis-related proteins (Scherer et al., 2005) and host immune response proteins (Urwin et al., 2002). Genes linked to reproduction are also frequently a target of diversifying selection (Swanson and Vacquier, 2002). Positive selection is commonly identified after gene duplication, reflecting a process in which the new copies accumulate mutations that may result in functional evolutionary novelties. 

Considering the high level of diversification in the Solanaceae RNases T2, it would be interesting to test whether the positive selection is the primary evolutionary force leading to an increased amino acid variability in these proteins. Indeed, our group has demonstrated a statistically significant number of non-synonymous substitutions in the hypervariable and variable regions of *Solanum* S-RNase sequences (Brisolara-Corrêa et al., 2015). Other studies using smaller numbers and sets of S-RNases also identified some positively selected residues (Savage and Miller, 2006; Miller *et al.,* 2008; Paape and Kohn, 2011). In this study, we have now shown that RNase T2 proteins from the Solanaceae are also subjected to positive selection and that some positively selected amino acids are located in regions of functional importance (Table S5). Additionally, different sites are under positive selection in clade 3 and subclade 3 of clade 2, indicating that different residues contribute to the specificity of S-RNases and S-like RNases. 

S-RNases are involved in the SI system. They have two key hypervariable regions, HVa and HVb, which seem to be related to the determination of allelic identity (Li et al., 2017). HVa includes a long loop (Lys47-Thr56) that connects the C-terminal Asp37 of the β-strand β2 to the N-terminal Gly57 of the long α-helix α2. This flexible loop probably allows an allele-specific interaction with S-gene products. The side-chains of all amino acids in the HVa and HVb regions are solvent accessible. The catalytic acid and base are His32 and His91, respectively (Ida et al., 2001). Critical structural differences were found in S-RNases compared to S-like RNases, including differences in the structural conformation near the B2 substrate-binding site and a lack of stacking interactions (Tyr17-substrate-Phe89) typical of S-like RNases. We also observed differences in the electrostatic potential near the B1 and B2 substrate binding sites and the presence of residues predicted as positively selected in considerably more rigid regions. Finally, we have found S-RNases, for example, the 1IOO, to be more hydrophobic than S-like RNases (Figure S8), which could be related to the extracellular location of S-RNases and their interaction with membrane transporters (Williams et al., 2015).

The interaction between Tyr17 and Phe89 at the B2 site of RNases LE (1DIX), NW (1IYB), and NT (1VD1) seem to be responsible for the tighter binding of purine bases (A and G). Additionally, His39 and His97 are probably the acid and base catalysts, whereas His92 binds the phosphate group of the substrate (Tanaka et al., 2000, for review). Like RNase LE (1DIX), RNase NW (1IYB) has a preference for guanylic acid (Tanaka *et al.,* 2000; Kawano et al., 2002); the amino acids Gln12, Tyr17, Thr78, Leu79, and Phe89 located at the B2 site seem to be jointly responsible for the recognition of this molecule. The moieties for 5’-GMP and 5’-AMP binding at the B2 site have a similar amino acid composition in RNase NT and RNase NW. However, there are some differences in the hydrogen bonds. For example, RNase NT (1VD1) has a broad base specificity; it can hydrolyze Poly A, Poly I, and Poly U (Kawano *et al.,* 2006). These bases bind at the B1 site formed by Trp42, Asn44, and Trp50. In RNase NT, Trp50 creates a more hydrophobic pocket than Tyr50 in RNase NW and RNase LE. Consequently, it facilitates the binding of guanine, adenine, and uracil, which could explain the flexibility to recognize a broad spectrum of substrates (Kawano *et al.,* 2006).

We found that in a complex of S-RNase with 5’-GMP (Figure 3C), only a single hydrogen bond with the substrate is formed (involving Trp35), without the formation of the stacking sandwich (Tyr17-substrate-Phe89) typical of S-like RNases. This difference seems to be due to Ser83, which replaced Phe89 in the B2 binding site near 5’-GMP. Additionally, two positively selected residues (Phe72 and Pro82) are located near Gln69 in the B2 site. In the S-RNase complexed with 5’-AMP, the stacking interactions were also lost from the B1 and B2 binding sites, and a significantly smaller number of hydrogen bonds were formed. 

The electrostatic potential of S-RNase was also significantly different from that of S-like RNases. Li et al. (2017) demonstrated that the pistil S-RNase and the pollen S-locus F-box proteins (called SLF in Solanaceae) interact through attractive and repulsive forces that are a function of surface electrostatic potentials. They are responsible for the self/non-self discrimination between cytosolic proteins in *Petunia hybrida*. Our results indicate that the electrostatic potential plays an essential role in RNA degradation catalyzed by S-RNase.

In conclusion, our study represents the most wide-ranging and comprehensive analysis linking molecular evolution and structural aspects of S-RNases and S-like RNases in the Solanaceae to date. Future studies involving molecular dynamics simulations of S-RNases and S-like RNases complexed with different base substrates may contribute crucial clues to the behavior of essential residues and their connection to base specificity. 

## Supplementary material

The following online material is available for this article:

Table S1 - Ribonuclease sequences of the T2 gene family considered in the analyses.

Table S2 - Output information for the first five best-fit models in jModelTest using the DNA sequences and the first five best-fit models in ProtTest using the amino acid dataset.

Table S3 - Likelihood ratio test (LRT) of TPM3uf+G and GTR+G models.

Table S4 - Protein length, isoelectric point (pI), and molecular weight (MW) of clade 1 and 2 Solanaceae RNases T2.

Table S5 - Likelihood scores under the branch-site model of variable ω ratios among sites and branches, and positively selected sites (PSS) for T2 RNase genes in the Solanaceae family.

Figure S1 - Neighbor Joining genealogy based on the nucleotide coding sequence of Solanaceae RNases T2.

Figure S2 - Neighbor Joining genealogy based on the amino acid sequence of Solanaceae RNases T2.

Figure S3 - Extended Clade 3 illustrating the maximum likelihood genealogy based on the nucleotide coding sequences of Solanaceae S-RNases.

Figure S4 - Root mean square deviation (RMSD) for RNase C_α_ atoms over 120 ns of molecular dynamics simulation.

Figure S5 - Number of amino acid residues related to each secondary structure over the time of molecular dynamics simulations, obtained using the DSSP software.

Figure S6 - Secondary structure of the amino acid residues over the time of molecular dynamics simulations, obtained using the DSSP software.

Figure S7 - Root mean square fluctuation (RMSF) for RNase C_α_ atoms over the final five ns of molecular dynamics simulation.

Figure S8 - Hydrophilic (red), hydrophobic (black), and total (green) solvent accessible surface (SAS) of RNase structures after 120 ns of molecular dynamics simulation.

Figure S9 - Structure superposition of the catalytic residues.

## References

[B1] Aguiar B, Vieira J, Cunha AE, Vieira CP (2015). No evidence for Fabaceae gametophytic self-incompatibility being
determined by Rosaceae, Solanaceae, and Plantaginaceae S-RNase lineage
genes. BMC Plant Biol.

[B2] Altschul SF, Gish W, Miller W, Myers EW, Lipman DJ (1990). Basic local alignment search tool. J Mol Biol.

[B3] Anisimova M, Bielawski JP, Yang Z (2001). Accuracy and power of the likelihood ratio test in detecting
adaptive molecular evolution. Mol Biol Evol.

[B4] Anisimova M, Gascuel O (2006). Approximate likelihood-ratio test for branches: A fast, accurate,
and powerful alternative. Syst Biol.

[B5] Baker NA, Sept D, Joseph S, Holst MJ, McCammon JA (2001). Electrostatics of nanosystems: Application to microtubules and
the ribosome. Proc Natl Acad Sci U S A.

[B6] Bariola A, Howard CJ, Taylor CB, Verburg MT, Jagian VD, Green PJ (1994). The Arabidopsis ribonuclease gene RNS1 is tightly controlled in
response to phosphate limitation. Plant J.

[B7] Berendsen HJC, Grigera JR, Straatsma TP (1987). The missing term in effective pair potentials. J Phys Chem.

[B8] Bielawski JP, Yang Z, Nielsen R (2005). Statistical methods in molecular evolution.

[B9] Bishop JG (2005). Directed mutagenesis confirms the functional importance of
positively selected sites in polygalacturonase inhibitor
protein. Mol Biol Evol.

[B10] Brisolara-Corrêa L, Thompson CE, Fernandes CL, Freitas LB (2015). Diversification and distinctive structural features of S-RNases
alleles in the genus Solanum. Mol Genet Genomics.

[B11] Bussi G, Donadio D, Paarrinello M (2007). Canonical sampling through velocity rescaling. J Chem Phyt.

[B12] Chen G, Zhang B, Zhao Z, Sui Z, Zhang H, Xue Y (2010). ‘A life or death’ decision for pollen tubes in S0RNase-based
self-incompatibility. J Exp Bot.

[B13] Darriba D, Taboada GL, Doallo R, Posada D (2011). ProtTest 3: Fast selection of best-fit models of protein
evolution. Bioinformatics.

[B14] Darriba D, Taboada GL, Doallo R, Posada D (2012). jModelTest 2: More models, new heuristics and parallel
computing. Nat Methods.

[B15] Deshpande RA, Shankar V (2002). Ribonucleases from T2 family. Crit Rev Microbiol.

[B16] Dodds PN, Clarke AE, Newbigin E (1996). Molecular characterization of an S-like RNase of Nicotiana alata
that is induced by phosphate starvation. Plant Mol Biol.

[B17] Dolinsky TJ, Nielsen JE, McCammon JA, Baker NA (2004). PDB2PQR: An automated pipeline for the setup, execution, and
analysis of Poisson-Boltzmann electrostatics calculations. Nucleic Acids Res.

[B18] Ellegren H (2008). Comparative genomics and the study of evolution by natural
selection. Mol Ecol.

[B19] Entani T, Kubo K-I, Isogai S, Fukao Y, Shirakawa M, Isogai A, Takayama S (2014). Ubiquitin-proteasome-mediated degradation of S-RNase in a
solanaceous cross-compatibility reaction. Plant J.

[B20] Essmann U, Perera L, Berkowitz ML, Darden T, Lee H, Pedersen LG (1995). A smooth particle mesh Ewald method. J Chem Phys.

[B21] Felsenstein J (1985). Confidence limits on phylogenies: An approach using the
bootstrap. Evolution.

[B22] Goldstein DM, Shu S, Howson R, Neupane R, Hayes RD, Fazo J, Mitros T, Dirks W, Hellsten U, Putnam N (2012). Phytozome: A comparative platform for green plant
genomics. Nucleic Acids Res.

[B23] Green PJ (1994). The ribonucleases of higher plants. Annu Rev Plant Physiol.

[B24] Guindon S, Dufayard JF, Lefort V, Anisimova M, Hordijk W, Gascuel O (2010). New algorithms and methods to estimate maximum-likelihood
phylogenies: Assessing the performance of PhyML 3.0. Syst Biol.

[B25] Hayashi T, Kobayashi D, Kariu T, Tahara M, Hada K, Kouzuma Y, Kimura M (2003). Genomic cloning of ribonucleases in Nicotiana glutinosa leaves,
as induced in response to wounding or to TMV-infection, and characterization
of their promoters. Biosci Biotechnol Biochem.

[B26] Hess B, Bekker H, Berendsen HJC, Fraaije J (1997). Lincs: A linear constraint solver for molecular
simulations. J Comput Chem.

[B27] Hillwig MS, Liu X, Liu G, Thornburg RW, MacIntosh GC (2010). Petunia nectar proteins have ribonuclease
activity. J Exp Bot.

[B28] Hua Z-H, Fields A, Kao T-H (2008). Biochemical models for S-RNase-based
self-incompatibility. Mol Plant.

[B29] Ida K, Norioka S, Yamamoto M, Kumasaka T, Yamashita E, Newbigin E, Clarke AE, Sakiama F, Sato M (2001). The 1.55Å resolution structure of Nicotiana alata Sf11-RNase
associated with gametophytic self-incompatibility. J Mol Biol.

[B30] Igić B, Kohn JR (2001). Evolutionary relationships among self-incompatibility
RNases. Proc Natl Acad Sci U S A.

[B31] Irie M (1999). Structure-function relationships of acid ribonucleases:
Lysosomal, vacuolar, and periplasmic enzymes. Pharmacol Ther.

[B32] Iwano M, Takayama S (2012). Self/non-self discrimination in angiosperm
self-incompatibility. Curr Opin Plant Biol.

[B33] Kawano S, Kakuta Y, Kimura M (2002). Guanine binding site of the Nicotiana glutinosa ribonuclease NW
revealed by X-ray crystallography. Biochemistry.

[B34] Kawano S, Kakuta Y, Nakashima T, Kimura M (2006). Crystal structures of the Nicotiana glutinosa ribonuclease NT in
complex with nucleoside monophosphates. J Biochem.

[B35] Kawata Y, Sakiyama F, Tamaoki H (1998). Amino-acid sequence of ribonuclease T2 from Aspergillus
oryzae. Eur J Biochem.

[B36] Köch M, Stenzel I, Zimmer A (2006). Tissue-specific expression of tomato ribonuclease LX during
phosphate starvation-induced root growth. J Exp Bot.

[B37] Kubo K-I, Paape T, Hatakeyama M, Entani T, Takara A, Kajihara K, Tsukahara M, Shimizu-Inatsugi R, Shimizu KK, Takayama S (2015). Gene duplication and genetic exchange drive the evolution of
S-RNase-based self-incompatibility in Petunia. Nat Plants.

[B38] Kumar S, Stecher G, Li M, Knyaz C, Tamura K (2018). MEGA X: Molecular evolutionary genetics analysis across computing
platforms. Mol Biol Evol.

[B39] Kunitz M (1940). Crystalline ribonuclease. J Gen Physiol.

[B40] Kurata N, Kariu T, Kawano S, Kimura M (2002). Molecular cloning of cDNAs encoding ribonuclease-relates proteins
in Nicotiana glutinosa leaves, as induced in response to wounding or to
TMV-infection. Biosci Biotechnol Biochem.

[B41] Lee H-S, Huang S, Kao T-H (1994). S-proteins control rejection of incompatible pollen in Petunia
inflata. Nature.

[B42] Leeuw M, Roiz L, Smirnoff P, Schwartz B, Shoseyov O, Almog O (2007). Binding assays and preliminary X-ray crystallographic analysis of
ACTIBIND, a protein with anticarcinogenic and antiogiogenic
activities. Acta Crystallogr Sect F Struct Biol Cryst Commun.

[B43] Lers A, Khalchitski A, Lomaniec E, Burd S, Green PJ (1998). Senescence-induced RNases in tomato. Plant Mol Biol.

[B44] Li J, Zhang Y, Song Y, Zhang H, Fan J, Li Q, Zhang D, Xue Y (2017). Electrostatic potentials of S-locus F-box proteins contribute to
the pollen S specificity in self-incompatibility in Petunia
hybrida. Plant J.

[B45] Luhtala N, Parker R (2010). T2 family ribonucleases: Ancient enzymes with diverse
roles. Trends Biochem Sci.

[B46] MacIntosh GC, Hillwig MS, Meyer A, Flagel L (2010). RNase T2 genes from rice and the evolution of secretory
ribonucleases in plants. Mol Genet Genomics.

[B47] Matton DP, Nass N, Clarke AE, Newbigin E (1994). Self-incompatibility: How plants avoid illegitimate
offspring. Proc Natl Acad Sci U S A.

[B48] Miller JS, Levin RA, Feliciano MM (2008). A tale of two continents: Baker’s rule and the maintenance of
self-incompatibility in Lycium (Solanaceae). Evolution.

[B49] Miyamoto S, Kollman PA (1992). SETTLE: An analytical version of the SHAKE and RATTLE algorithm
for rigid water models. J Comp Chem.

[B50] Murfett J, Atherton TL, Mou B, Gasser CS, McClure BA (1994). S-RNase expressed in transgenic Nicotiana causes
S-allele-specific pollen rejection. Nature.

[B51] Oostenbrink C, Villa A, Mark AE, van Gunsteren WF (2004). A biomolecular force field based on the free enthalpy of
hydration and solvation: The GROMOS force-field parameter sets 53A5 and
53A6. J Comput Chem.

[B52] Paape T, Kohn JR (2011). Differential strengths of selection on S-RNases from Physalis and
Solanum (Solanaceae). BMC Evol Biol.

[B53] Papadopoulos JS, Agarwala R (2007). COBALT: Constraint-based alignment tool for multiple protein
sequences. Bioinformatics.

[B54] Parrinello M, Rahman A (1981). Polymorphic transitions in single crystals: A new molecular
dynamics method. J Appl Phys.

[B55] Pretz C, Smith SD (2022). Intraspecific breakdown of self-incompatibility in Physalis
acutifolia (Solanaceae). AoB Plants.

[B56] Ramanauskas K, Igić B (2017). The evolutionary history of plant T2/S-type
ribonucleases. PeerJ.

[B57] Roalson EH, McCubbin AG (2003). S-RNases and sexual incompatibility: Structure, functions, and
evolutionary perspectives. Mol Phylogenet Evol.

[B58] Savage AE, Miller JS (2006). Gametophytic self-incompatibility in Lycium parishii
(Solanaceae): Allelic diversity, genealogical structure, and patterns of
molecular evolution. Heredity (Edinb).

[B59] Scherer NM, Thompson CE, Freitas LB, Bonatto SL, Salzano FM (2005). Patterns of molecular evolution in pathogenesis-related
proteins. Genet Mol Biol.

[B60] Singh A, Ai Y, Kao T-H (1991). Characterization of ribonuclease activity of three
S-allele-associated proteins of Petunia inflata. Plant Physiol.

[B61] Steinbachs JE, Holsinger KE (2002). S-RNase-mediated gametophytic self-incompatibility is ancestral
in eudicots. Mol Biol Evol.

[B62] Swanson WJ, Vacquier VD (2002). The rapid evolution of reproductive proteins. Nat Rev Genet.

[B63] Takayama S, Isogai A (2005). A self-incompatibility in plants. Annu Rev Plant Biol.

[B64] Tanaka N, Arai J, Inokuchi N, Koyama T, Ohgi K, Irie M, Nakamura KT (2000). Crystal structure of a plant ribonuclease. RNase LE. J Mol Biol.

[B65] Torres-Rodríguez MD, Cruz-Zamora Y, Juárez-Díaz JA, Mooney B, McClure BA, Cruz-García F (2020). NaTrxh is an essential protein for pollen rejection in Nicotiana
by increasing S-RNase activity. Plant J.

[B66] Touw WG, Baakman C, Black J, te Beek TAH, Krieger E, Joosten RP, Vriend G (2015). A series of PDB-related databases for everyday
needs. Nucleic Acids Res.

[B67] Urwin R, Holmes EC, Fox AJ, Derrick JP, Maiden MCJ (2002). Phylogenetic evidence for frequent positive selection and
recombination in the meningococcal surface antigen PorB. Mol Biol Evol.

[B68] van der Spoel D, Lindahl E, Hess B, Groenhof G, Mark AE, Berendsen HJC (2005). Gromacs: Fast, flexible, and free. J Comput Chem.

[B69] Williams JS, Wu L, Li S, Sun P, Kao T-H (2015). Insight into S-RNase-based self-incompatibity in Petunia: Recent
findings and future directions. Front Plant Sci.

[B70] Wong WSW, Yang Z, Goldman N, Nielsen R (2004). Accuracy and power of statistical methods for detecting adaptive
evolution in protein coding sequences and for identifying positively
selected sites. Genetics.

[B71] Wu L, Xu Y, Zhao H, Li Y (2020). RNase T2 in inflammation and cancer: Immunological and biological
views. Front Immunol.

[B72] Xu B, Yang Z (2013). PAMLX: A graphical user interface for PAML. Mol Biol Evol.

