# Supplementary material for: Evolutionary and structural aspects of Solanaceae RNases T2
Source: Genet Mol Biol. 2022 Dec 16;46(1 Suppl 1):e20220115. doi: 10.1590/1678-4685-GMB-2022-0115 (PMC9762611; doi:10.1590/1678-4685-GMB-2022-0115)
Supplement: Table S1 - [file 1415-4757-GMB-46-1-s1-e20220115-s1.pdf]

Genetics and Molecular Biology
DOI: https://doi.org/10.1590/1678-4685-GMB-2022-0115

Supplementary Material to "Evolutionary and structural aspects of Solanaceae RNases T2"

Table S1. Ribonuclease sequences from T2 gene family considered in the analyses, species from which they were obtained, OTU names, clustering information, and NCBI accessions numbers.

Species	OTU	C[a]	NCBI[b]
Solanum tuberosum	Stuberosum_3I	1	565353567
Solanum lycopersicum	Slycopersicum_LX1	1	460394145
Solanum tuberosum	Stuberosum_2I	1	565353565
Solanum tuberosum	Stuberosum_S3	1	565353259
Nicotiana alata	Nalata_NE	2_2	532754
Nicotiana glutinosa	Nglutinosa_1	2_2	31621002
Solanum lycopersicum	Slycopersicum_LE1	2_2	350535451
Solanum lycopersicum	Lesculentum_LE	2_2	4582640
Solanum tubersosum	Stuberosum_LE	2_2	565377256
Nicotiana tomentosiformis	Ntomentosiformis	2_2	697144165
Solanum lycopersicum	Slycopersicum_LE2	2_1	723698815
Solanum tuberosum	Stuberosum_S1	2_1	565354869
Solanum tuberosum	Stuberosum_LX	2_1	565354740
Solanum lycopersicum	Slycopersicum_LE3	2_1	460382631
Solanum lycopersicum	Lesculentum_LX	2_3	4582642
Solanum lycopersicum	Slycopersicum_LX	2_3	350537479
Solanum tuberosum	Stuberosum_LX2	2_3	565377258
Solanum tuberosum	Stuberosum_1	2_3	88683128
Nicotiana glutinosa	Nglutinosa_3	2_3	5902456
Petunia hybrida	Phybrida_5	2_3	258617482
Lycium bosciifolium	LbosciifoliumS10	3	323320192
Lycium pumilum	LpumilumS3	3	323320214
Lycium oxycarpum	Loxycarpum S3	3	158516104
Lycium pumilum	LpumilumS7	3	323320222
Lycium hirsutum	LhirsutumS12	3	323320210
Lycium hirsutum	LhirsutumS3	3	158516098
Lycium pumilum	LpumilumS12	3	323320230
Lycium ferocissimum	LferocissimumS5	3	158516092
Nicotiana sylvestris	Nsylvestris	3	2578426
Petunia axillaris	PaxillarisS1	3	13194187
Petunia hybrida	PhybridaS11	3	311334657
Nicotiana alata	NalataS70	3	295883693
Nicotiana alata	Nalata1	3	2696958

Species	OTU	C[a]	NCBI[b]
Nicotiana alata	NalataS63	3	295883705
Petunia hybrida	PhybridaS7	3	311334655
Nicotiana glauca	NglaucaS5	3	56067047
Nicotiana alata	NalataS5	3	295883695
Nicotiana alata	NalataS75	3	295883697
Nicotiana glauca	NglaucaS3S8	3	56067049
Lycium cestroides	LcestroidesSc	3	158516056
Lycium parishii	LyparishiiS5	3	86991384
Solanum chacoense	Schacoense2	3	288519
Solanum chilense	SchilenseS24	3	157377666
Lycium parishii	LparishiiS9	3	86991392
Eriolarynx lorentzii	ElorentziiS2	3	166237167
Solanum peruvianum	SperuvianumSP16	3	313247956
Iochroma cyaneum	IcyaneumS1	3	166237179
Iochroma gesnerioides	IgesnerioidesS2	3	166237183
Solanum chacoense	SchacoenseS12	3	5919069
Lycium cestroides	LcestroidesSg	3	323320294
Vassobia breviflora	VbrevifloraS1	3	166237185
Solanum habrochaites	Shabrochaites5	3	288548534
Solanum habrochaites	Shabrochaites4	3	288548532
Solanum chilense	SchilenseS6	3	157377676
Solanum peruvianum	SperuvianumS11a	3	443777
Lycium andesonii	LandensoniiS1	3	4235023
Brugmansia versicolor	BversicolorS1	3	56067051
Nicotiana alata	Nalata_2	3	2696960
Dunalia brachyantha	DbrachyanthaS1	3	166237171
Iochroma australe	IaustraleS1	3	166237173
Eriolarynx lorentzii	ElorentziiS1	3	166237165
Iochroma gesnerioides	IgesnerioidesS1	3	166237181
Nicotiana alata	Nalata_S3	3	1519368
Vassobia breviflora	VbrevifloraS2	3	166237187
Brugmansia versicolor	BversicolorS2	3	56067053
Witheringia maculata	WmaculataS5	3	6684285
Witheringia maculata	WmaculataS2	3	6684286
Physalis longifolia	PlongifoliaS18	3	14456326
Physalis longifolia	PlongifoliaS22	3	14456330
Physalis cinerascens	PcinerascensS11	3	4160428
Physalis crassifolia	PcrassifoliaS21	3	1146378
Physalis longifolia	PlongifoliaS9	3	14456312
Witheringia maculata	WmaculataS4	3	6684288
Witheringia maculata	WmaculataS11	3	6684289
Physalis longifolia	PlongifoliaS27	3	14456338
Physalis longifolia	PlongifoliaS33	3	20385645
Solanum lycopersicum	SlyZS11	3	343457989

Species	OTU	C[a]	NCBI[b]
Solanum lycopersicum	SlycopersicumZS12	3	343457991
Solanum habrochaites	ShabrochaitesZS8	3	343458007
Solanum peruvianum	SperuvianumS13	3	443783
Solanum lycopersicum	SlycopersicumS18	3	379997145
Solanum chilense	SchilenseZS9	3	345462632
Solanum peruvianum	SperuvianumS7	3	404317
Solanum habrochaites	Shabrochaites3	3	288548530
Petunia hybrida	PhybridaSx2	3	169250
Petunia hybrida	PhybridaSo	3	253796266
Petunia axillaris	PaxillarisS19	3	59896629
Lycium ferocissimum	LferocissimumS8	3	323320200
Physalis longifolia	PlongifoliaS20	3	14456328
Lycium parishii	LparishiiS15	3	86991404
Solanum chilense	SchilenseS17	3	157377708
Solanum peruvianum	SperuvianumSP7	3	313247942
Petunia axillaris	PaxillarisS13	3	13194189
Nicotiana alata	NalataS210	3	295883703
Lycium andersonii	LandersoniiS2	3	4235025
Petunia inflata	PinflataS3	3	84778495
Petunia axillaris	PaxillariaS15	3	13194191
Petunia hybrida	PhybridaSx1	3	169248
Petunia axillaris	PaxillarisSC2	3	26225031
Solanum carolinense	ScarolinenseESC	3	1161186
Solanum carolinense	ScarolinenseSV	3	57545733
Solanum chilense	SchilenseS30	3	157377704
Solanum habrochaites	Shabrochaites2	3	288548528
Solanum chilense	SchilenseS21	3	157377702
Solanum chilense	SchilenseS3	3	157377710
Solanum stenotomum	SstenotomumS1	3	344222023
Solanum carolinense	ScarolinenseSR	3	57545741
Solanum chilense	SchilenseS22	3	157377706
Solanum neorickii	SneorickiiS1	3	21623713
Solanum peruvianum	SperuvianumS24	3	21623696
Solanum habrochaites	Shabrochaites1	3	288548526
Solanum chilense	SchilenseS1	3	21623701
Solanum peruvianum	SperuvianumS15	3	21623679
Physalis crassifolia	PcrassifoliaS14	3	1146362
Physalis crassifolia	PcrassifoliaS6	3	1146400
Physalis crassifolia	PcrassifoliaS3	3	1146394
Witheringia maculata	WmaculataS7	3	6684290
Physalis crassifolia	PcrassifoliaS16	3	1146366
Physalis crassifolia	PcrassifoliaS5	3	1146398
Physalis longifolia	PlongifoliaS7	3	14456308
Physalis longifolia	PlongifoliaS8	3	14456310

Species	OTU	C[a]	NCBI[b]
Physalis crassifolia	PcrassifoliaS11	3	1146356
Physalis cinerascens	PcinerascensS6	3	4160418
Physalis longifolia	PlongifoliaS10	3	20385633
Physalis cinerascens	PcinerascensS9	3	4160424
Physalis longifolia	PlongifoliaS11	3	20385635
Solanum chacoense	Schacoense1	3	7110526
Solanum chilense	SchilenseZS4	3	343457999
Solanum chilense	SchilenseZS6	3	343458003
Solanum chilense	SchilenseS15	3	352962790
Solanum chilense	SchilenseS16	3	352962792
Lycium bosciifolium	LbosciifoliumS4	3	158516070
Lycium ferocissimum	LferocissimumS1	3	158516084
Lycium parishii	LparishiiS19	3	86991410
Lycium truncatum	LtruncatumS6	3	323320181
Lycium schweinfurthii	LschweinfurthiiS7	3	323320280
Lycium bosciifolium	LbosciifoliumS3	3	158516068
Lycium parishii	LparishiiS6	3	86991386
Lycium chinense	LchilenseS7	3	323320172
Lycium parishii	LparishiiS21	3	86991412
Lycium parishii	LparishiiS22	3	86991414
Lycium parishii	LparishiiS25	3	86991420
Lycium barbarum	LbarbarumS2	3	323320148
Lycium barbarum	LbarbarumS7	3	323320158
Lycium barbarum	LbarbarumS8	3	323320160
Lycium truncatum	LtruncatumS3	3	323320179
Lycium oxycarpum	LoxycarpumS10	3	323320238
Lycium ruthenicum	LruthenicumS1	3	323320177
Lycium parishii	LparishiiS23	3	86991416

[a]Major clades in the phylogeny are identified. The subclades are indicated after the underline symbol.
[b]NCBI accession numbers of the sequences used in the analysis.
The nomenclature of genes and alleles are those from the repository information and as originally reported in the related publications.
