# Supplementary material for: Evolutionary and structural aspects of Solanaceae RNases T2
Source: Genet Mol Biol. 2022 Dec 16;46(1 Suppl 1):e20220115. doi: 10.1590/1678-4685-GMB-2022-0115 (PMC9762611; doi:10.1590/1678-4685-GMB-2022-0115)
Supplement: Table S2 - [file 1415-4757-GMB-46-1-s1-e20220115-s2.pdf]

Genetics and Molecular Biology
DOI: https://doi.org/10.1590/1678-4685-GMB-2022-0115

Supplementary Material to "Evolutionary and structural aspects of Solanaceae RNases T2"

Table S2. Output information for the first five best-fit models in jModelTest using the DNA sequences and for the first five best-fit models in ProtTest using the amino acid dataset.

**jModelTest**	**1st model**	**2nd model**	**3rd model**	**4th model**	**5th model**	**Alpha(G)***
**DT**	TPM3uf+G	TIM3+G	GTR+G	TPM3+G	F81+G	1.31
**AICc**	TPM3uf+G	TVM+G	TIM3+G	GTR+G	HKY+G	1.29
**BIC**	TPM3uf+G	TVM+G	TIM3+G	GTR+G	HKY+G	1.29
**ProtTest**	**1st model**	**2nd model**	**3rd model**	**4th model**	**5th model**	**Alpha(G)***
**AIC**	JTT+G+F	WAG+G+F	WAG+G	JTT+G	CpREV+G	1.16
**AICc**	WAG+G	JTT+G	CpREV+G	LG+G	HIVb+G	1.30
**BIC**	JTT+G+F	WAG+G+F	WAG+G	JTT+G	CpREV+G	1.16
**ML**	JTT+G+F	WAG+G+F	WAG+G	JTT+G	CpREV+G	---

*Alpha and Frequencies are parameters estimated for G and F, respectively.
