# Supplementary material for: Evolutionary and structural aspects of Solanaceae RNases T2
Source: Genet Mol Biol. 2022 Dec 16;46(1 Suppl 1):e20220115. doi: 10.1590/1678-4685-GMB-2022-0115 (PMC9762611; doi:10.1590/1678-4685-GMB-2022-0115)
Supplement: Table S3 - [file 1415-4757-GMB-46-1-s1-e20220115-s3.pdf]

Genetics and Molecular Biology
DOI: https://doi.org/10.1590/1678-4685-GMB-2022-0115

Supplementary Material to "Evolutionary and structural aspects of Solanaceae RNases T2"

Table S3. Likelihood ratio test (LRT) between the TPM3uf+G and GTR+G models.

Null model	Alternative model	Degrees of freedom	LRT (δ)	*P*-value
TPM3uf +G	GTR+G	3	10.32	> 0.05

The parameter being tested is assumed by the current null model but not the alternative model. The null model is rejected when the *P*-value of the LRT is smaller than 0.01 using a χ^2 or mixed χ^2 distribution.
