# Supplementary material for: Evolutionary and structural aspects of Solanaceae RNases T2
Source: Genet Mol Biol. 2022 Dec 16;46(1 Suppl 1):e20220115. doi: 10.1590/1678-4685-GMB-2022-0115 (PMC9762611; doi:10.1590/1678-4685-GMB-2022-0115)
Supplement: Table S4 - [file 1415-4757-GMB-46-1-s1-e20220115-s4.pdf]

Genetics and Molecular Biology
DOI: https://doi.org/10.1590/1678-4685-GMB-2022-0115

Supplementary Material to “Evolutionary and structural aspects of Solanaceae RNases T2”

Table S4. Protein length, isoelectric point (pI) and molecular weight (MW) of Solanaceae RNases T2 of clades 1 and 2.

Species	OTU	Lenght	pI	MW
Solanum tuberosum	Stuberosum_3I	226	9.25	25665.93
Solanum lycopersicum	Slycopersicum_LX1	243	8.86	27109.53
Solanum tuberosum	Stuberosum_2I	285	8.75	32147.28
Solanum tuberosum	Stuberosum_S3	251	4.76	29111.37
Nicotiana alata	Nalata_NE	231	4.81	25426.36
Nicotiana glutinosa	Nglutinosa_1	229	4.63	25177.03
Solanum lycopersicum	Slycopersicum_LE1	230	4.57	25331.32
Solanum lycopersicum	Lesculentum_LE	198 (partial)	4.68	22024.51
Solanum tubersosum	Stuberosum_LE	230	4.74	25496.48
Nicotiana tomentosiformis	Ntomentosiformis	229	4.56	25250.04
Solanum lycopersicum	Slycopersicum_LE2	139 (partial)	6.18	16075.31
Solanum tuberosum	Stuberosum_S1	236	5.84	26636.62
Solanum tuberosum	Stuberosum_LX	240	6.15	27194.01
Solanum lycopersicum	Slycopersicum_LE3	240	6.64	27231.13
Solanum lycopersicum	Lesculentum_LX	233	5.96	26489.91
Solanum lycopersicum	Slycopersicum_LX	237	5.73	27018.43
Solanum tuberosum	Stuberosum_LX2	237	5.55	26956.26
Solanum tuberosum	Stuberosum_1	182 (partial)	5.65	20794.44
Nicotiana glutinosa	Nglutinosa_3	236	5.07	27048.37
Petunia hybrida	Phybrida_5	168 (partial)	5.16	18979.14
