# Supplementary material for: Evolutionary and structural aspects of Solanaceae RNases T2
Source: Genet Mol Biol. 2022 Dec 16;46(1 Suppl 1):e20220115. doi: 10.1590/1678-4685-GMB-2022-0115 (PMC9762611; doi:10.1590/1678-4685-GMB-2022-0115)
Supplement: Table S5 - [file 1415-4757-GMB-46-1-s1-e20220115-s5.pdf]

Genetics and Molecular Biology
DOI: https://doi.org/10.1590/1678-4685-GMB-2022-0115

Supplementary Material to "Evolutionary and structural aspects of Solanaceae RNases T2"

Table S5. Likelihood scores under the branch-site model of variable ω ratios among sites and branches, and positively selected sites (PSS) for T2 RNase genes in Solanaceae family.

Foreground branch	Model	lnL	$2\Delta\ell$ (df, P-value)	PSS residues in BEB (NEB)
1	A	-29401.87		Not allowed
	A'	-29400.30	3.14 (1, 0.0764)	---
2	A	-29403.93		Not allowed
	A'	-29401.82	4.22 (1, 0.0400)	---
3	A	-29401.52		Not allowed
	A'	-29389.94	23.16 (1, <0.0001)	55, 64, 149 (55, 64, 149, 179)
4	A	-29404.36		Not allowed
	A'	-29404.36	0.00 (1, 1.0000)	---
5	A	-29404.19		Not allowed
	A'	-29399.76	8.86 (1, 0.0029)	---
6	A	-29402.98		Not allowed
	A'	-29398.29	9.38 (1, 0.0022)	104 (6, 10, 11, 104)

A is used as a label for the branch-site null model (3 free parameters) and A' is used to designated the branch-site alternative model (4 free parameters, p_0, p_1, ω_0, ω_2)
