# Supplementary material for: Evolutionary and structural aspects of Solanaceae RNases T2
Source: Genet Mol Biol. 2022 Dec 16;46(1 Suppl 1):e20220115. doi: 10.1590/1678-4685-GMB-2022-0115 (PMC9762611; doi:10.1590/1678-4685-GMB-2022-0115)
Supplement: Figure S4 - [file 1415-4757-GMB-46-1-s1-e20220115-s9.pdf]

*Genetics and Molecular Biology*
DOI: https://doi.org/10.1590/1678-4685-GMB-2022-0115

# Supplementary Material to "Evolutionary and structural aspects of Solanaceae RNases T2"

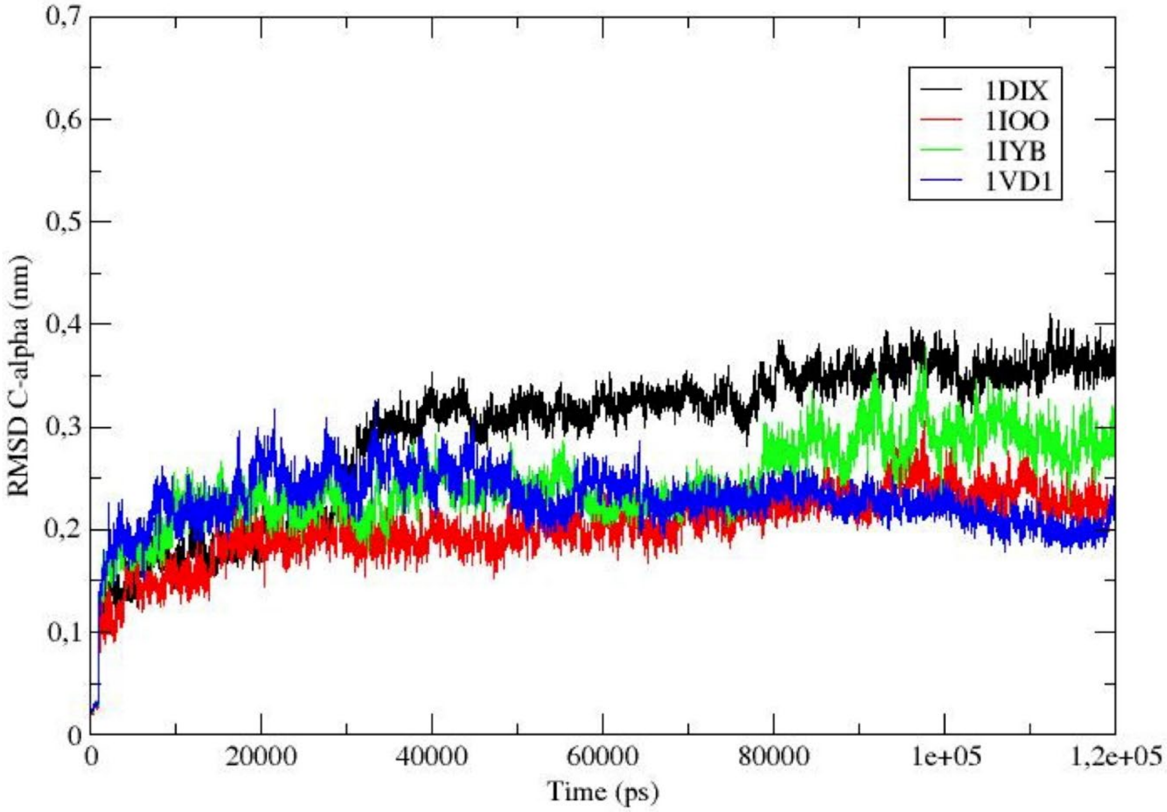


**Figure S4** - Root mean square deviation (RMSD) for RNase $C_\alpha$ atoms over 120 ns of molecular dynamics simulation considering the tested RNases 1DIX (black), 1IOO (red), 1IYB (green), and 1VD1 (blue).
