# Supplementary material for: Evolutionary and structural aspects of Solanaceae RNases T2
Source: Genet Mol Biol. 2022 Dec 16;46(1 Suppl 1):e20220115. doi: 10.1590/1678-4685-GMB-2022-0115 (PMC9762611; doi:10.1590/1678-4685-GMB-2022-0115)
Supplement: Figure S5 - [file 1415-4757-GMB-46-1-s1-e20220115-s10.pdf]

Genetics and Molecular Biology
DOI: https://doi.org/10.1590/1678-4685-GMB-2022-0115

Supplementary Material to "Evolutionary and structural aspects of Solanaceae RNases T2"

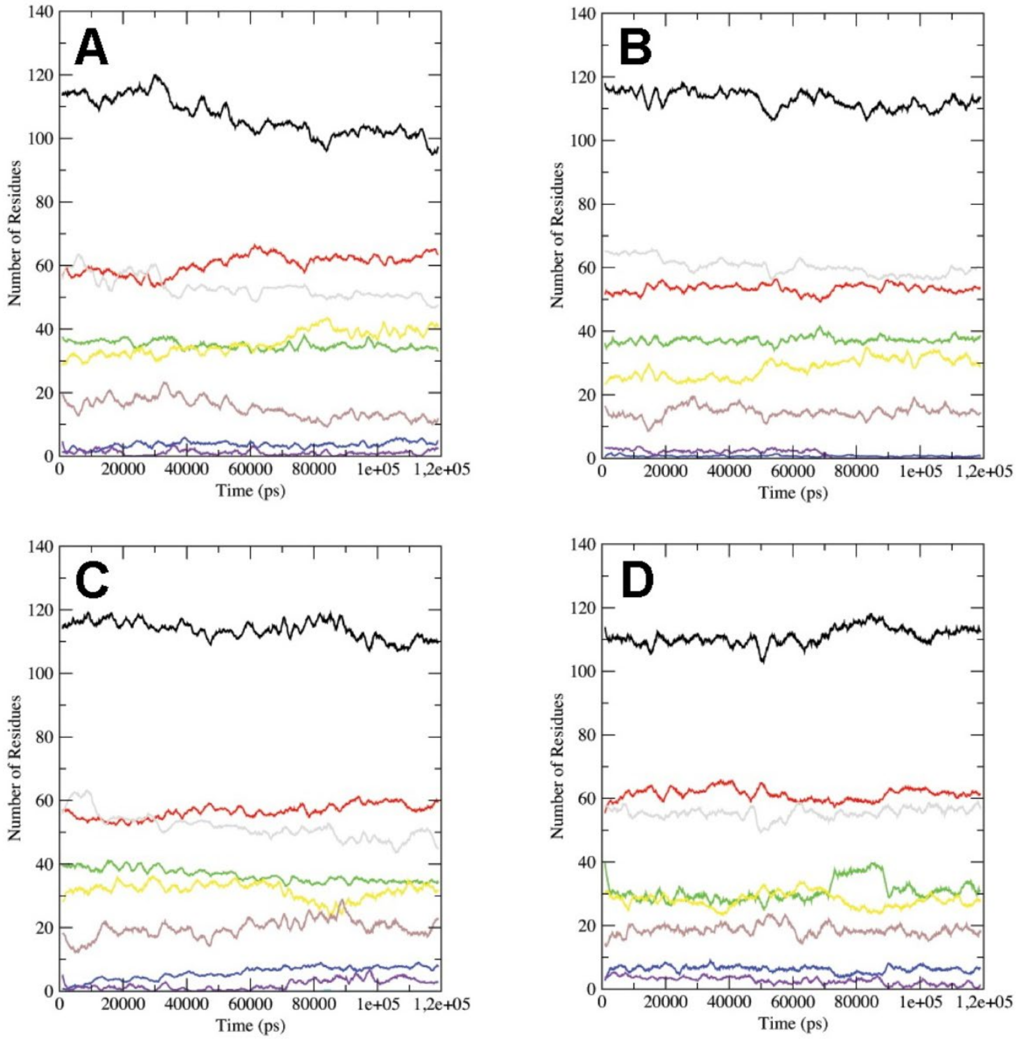

Figure S5 - Number of amino acid residues related to each secondary structure over the time of molecular dynamics simulations, obtained using the DSSP software: (A) 1DIX, (B) 1IOO, (C) 1IYB, and (D) 1VD1. Each color in the graphics corresponds to a specific secondary structure: black = structure; red = coil; green = β-sheet; blue = β-bridge; yellow = bend; pink = turn; light gray = A-helix; purple = 5-helix; and light blue = 3-helix.
