# Supplementary material for: Evolutionary and structural aspects of Solanaceae RNases T2
Source: Genet Mol Biol. 2022 Dec 16;46(1 Suppl 1):e20220115. doi: 10.1590/1678-4685-GMB-2022-0115 (PMC9762611; doi:10.1590/1678-4685-GMB-2022-0115)
Supplement: Figure S6 - [file 1415-4757-GMB-46-1-s1-e20220115-s11.pdf]

Genetics and Molecular Biology
DOI: https://doi.org/10.1590/1678-4685-GMB-2022-0115

Supplementary Material to "Evolutionary and structural aspects of Solanaceae RNases T2"

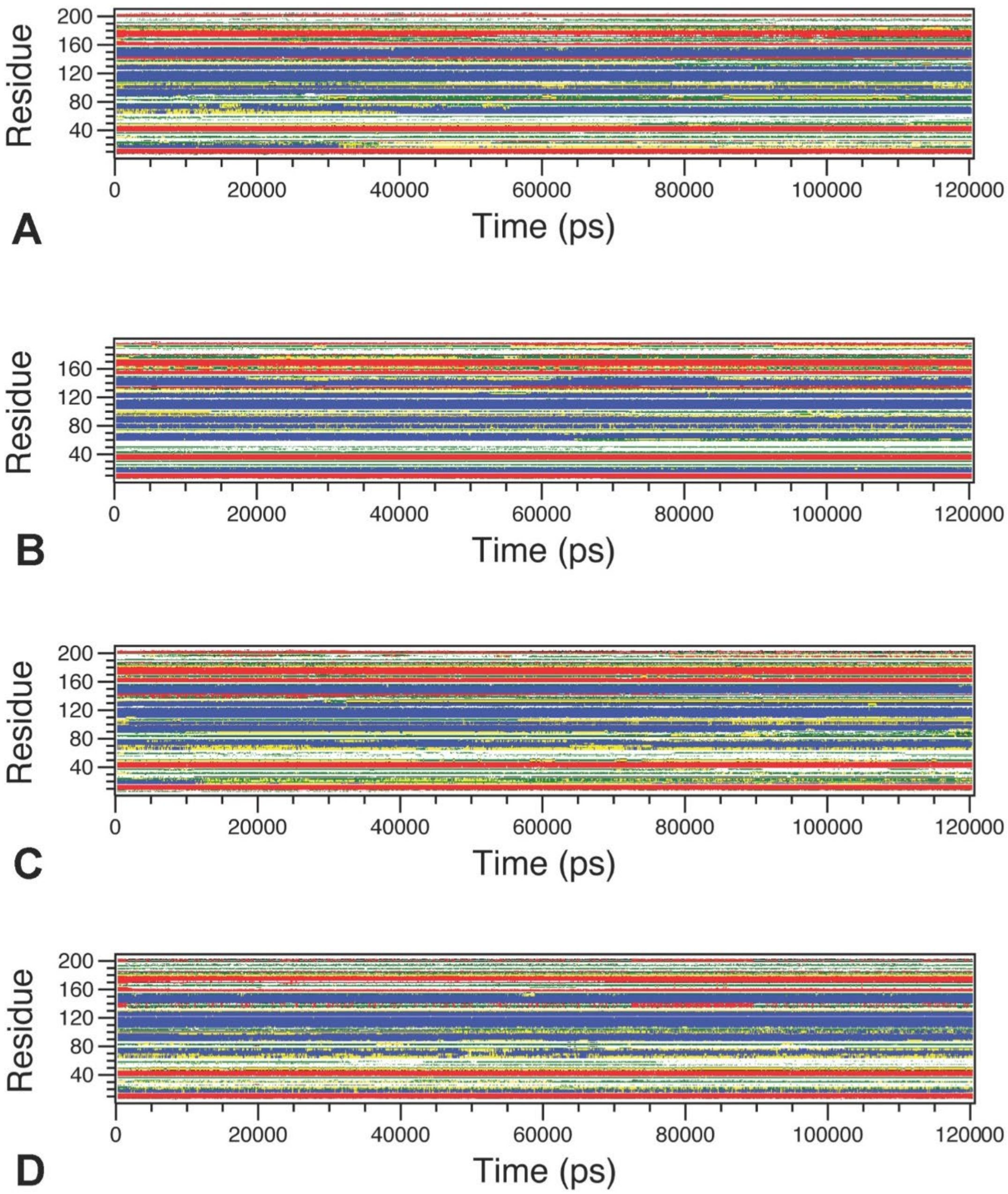

Figure S6 - Secondary structure of the amino acid residues over the time of molecular dynamics simulations, obtained using the DSSP software. (A) 1DIX, (B) 1IOO, (C) 1IYB, and (D) 1VD1. Each color in the graphics corresponds to a specific secondary structure: black = structure; red = coil; green = β-sheet; blue = β-bridge; yellow = bend; pink = turn; light gray = A-helix; purple = 5-helix; and light blue = 3-helix.
