# Supplementary material for: Evolutionary and structural aspects of Solanaceae RNases T2
Source: Genet Mol Biol. 2022 Dec 16;46(1 Suppl 1):e20220115. doi: 10.1590/1678-4685-GMB-2022-0115 (PMC9762611; doi:10.1590/1678-4685-GMB-2022-0115)
Supplement: Figure S7 - [file 1415-4757-GMB-46-1-s1-e20220115-s12.pdf]

Genetics and Molecular Biology
DOI: https://doi.org/10.1590/1678-4685-GMB-2022-0115

Supplementary Material to "Evolutionary and structural aspects of Solanaceae RNases T2"

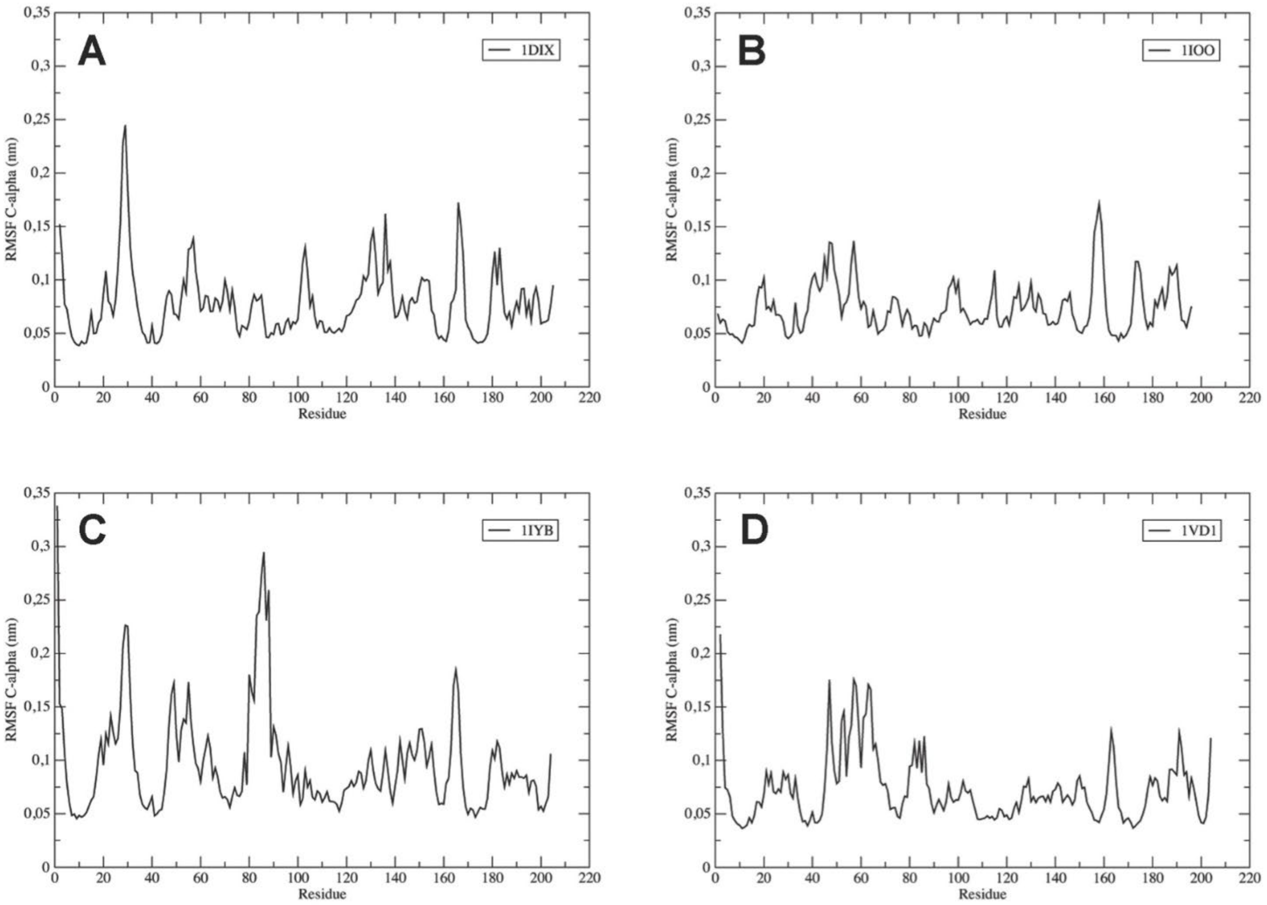

Figure S7 - Root mean square fluctuation (RMSF) for RNase C_α atoms over the final five ns of molecular dynamics simulation. (A) 1DIX, (B) 1IOO, (C) 1IYB, and (D) 1VD1.
