# Supplementary material for: Evolutionary and structural aspects of Solanaceae RNases T2
Source: Genet Mol Biol. 2022 Dec 16;46(1 Suppl 1):e20220115. doi: 10.1590/1678-4685-GMB-2022-0115 (PMC9762611; doi:10.1590/1678-4685-GMB-2022-0115)
Supplement: Figure S9 - [file 1415-4757-GMB-46-1-s1-e20220115-s14.pdf]

Genetics and Molecular Biology
DOI: https://doi.org/10.1590/1678-4685-GMB-2022-0115

Supplementary Material to "Evolutionary and structural aspects of Solanaceae RNases T2"

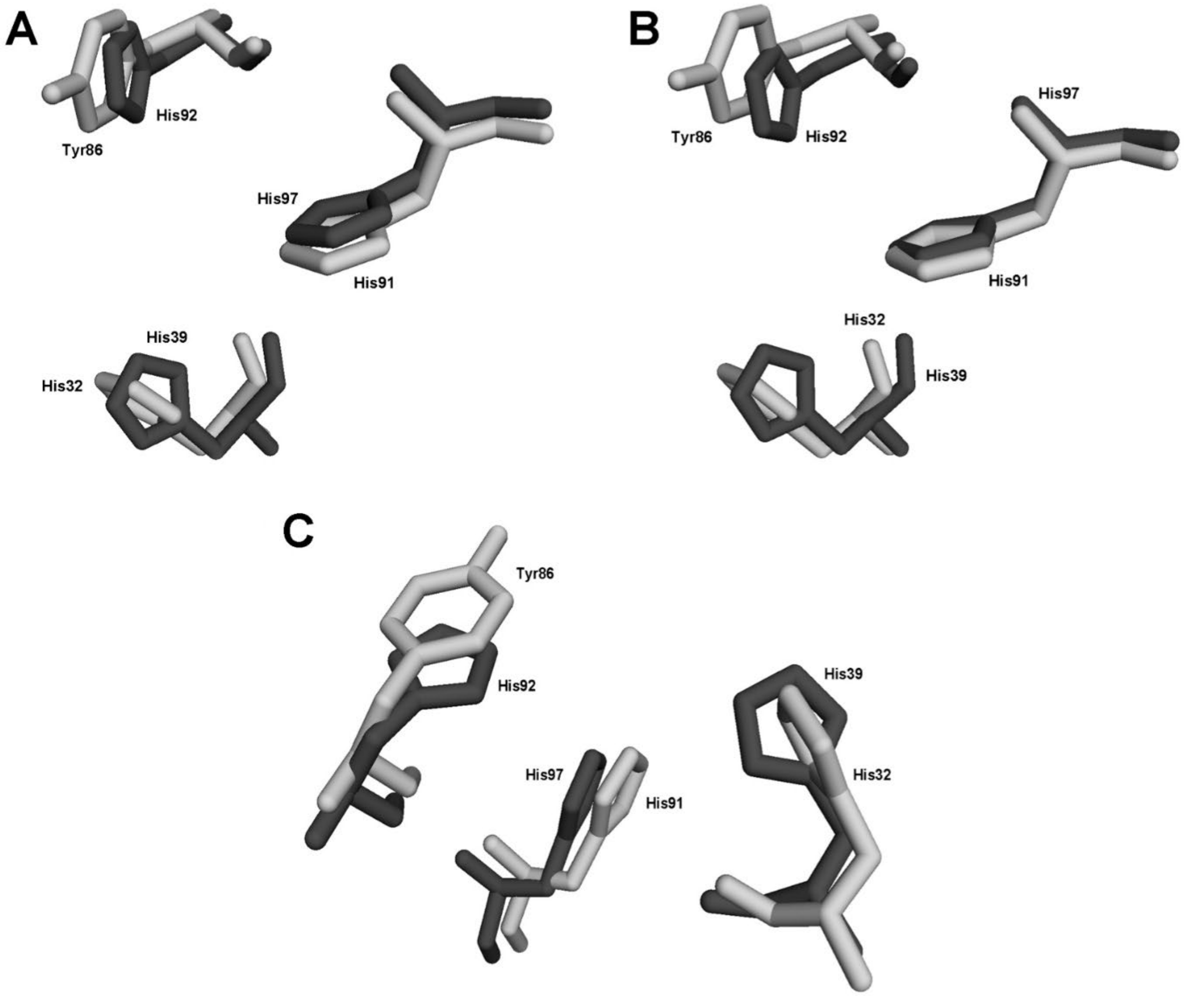

Figure S9 - Structure superposition of the catalytic residues: (A) S-RNase (1IOO) and S-like RNase LE (1DIX); (B) S-RNase (1IOO) and S-like RNase NW (1IYB); and (C) S-RNase (1IOO) and S-like RNase NT (1VD1).
